# Oxidative Stress and Inflammation: Drivers of Tumorigenesis and Therapeutic Opportunities

**DOI:** 10.3390/antiox14060735

**Published:** 2025-06-15

**Authors:** Meimei Wang, Yaping Xiao, Jie Miao, Xin Zhang, Meng Liu, Longchao Zhu, Hongxin Liu, Xiaoyan Shen, Jihui Wang, Biao Xie, Di Wang

**Affiliations:** 1 School of Life and Health Technology, Dongguan University of Technology, Dongguan 523808, China; mmwang@dgut.edu.cn (M.W.); xiaoyaping@dgut.edu.cn (Y.X.); 241222007@dgut.edu.cn (J.M.); 2023130@dgut.edu.cn (X.Z.); mliu@dgut.edu.cn (M.L.); zlc@dgut.edu.cn (L.Z.); liuhongxin@dgut.edu.cn (H.L.); xyshen@dgut.edu.cn (X.S.); wangjihui@dgut.edu.cn (J.W.); 2 Department of Gastroenterology, Guangzhou Eighth People’s Hospital, Guangzhou Medical University, Guangzhou 510440, China

**Keywords:** oxidative stress, inflammation, cancer biology, tumor therapies

## Abstract

As two pivotal regulatory factors in cancer biology, oxidative stress and inflammation interact dynamically through complex network mechanisms to influence tumor initiation, progression, and treatment resistance. Oxidative stress induces genomic instability, oncogenic signaling activation, and tumor microenvironment (TME) remodeling via the abnormal accumulation of reactive oxygen species (ROS) or reactive nitrogen species (RNS). Conversely, inflammation sustains malignant phenotypes by releasing pro-inflammatory cytokines and chemokines and promoting immune cell infiltration. These processes create a vicious cycle via positive feedback loops whereby oxidative stress initiates inflammatory signaling, while the inflammatory milieu further amplifies ROS/RNS production, collectively promoting proliferation, migration, angiogenesis, drug resistance, and immune evasion in tumor cells. Moreover, their crosstalk modulates DNA damage repair, metabolic reprogramming, and drug efflux pump activity, significantly impacting the sensitivity of cancer cells to chemotherapy, radiotherapy, and targeted therapies. This review systematically discusses these advances and the molecular mechanisms underlying the interplay between oxidative stress and inflammation in cancer biology. It also explores their potential as diagnostic biomarkers and prognostic indicators and highlights novel therapeutic strategies targeting the oxidative stress–inflammation axis. The goal is to provide a theoretical framework and translational roadmap for developing synergistic anti-tumor therapies.

## 1. Introduction

Cancer remains a formidable challenge to global health, with its intricate etiology and heterogeneous nature posing significant obstacles to effective treatment [1]. Among various factors driving tumor occurrence and progression, oxidative stress and inflammation have emerged as two pivotal regulatory factors that interact dynamically through intricate network mechanisms, which significantly influence tumor initiation, progression, and therapeutic resistance [2,3]. To achieve efficient cancer treatment, it is essential to understand their interplay.

Oxidative stress, an imbalance between reactive ROS/RNS and antioxidant defenses, plays a dual role in cancer biology. At low levels, ROS act as signaling molecules, modulating cell proliferation and survival via pathways like NF-κB and mitogen-activated protein kinase (MAPK) [4,5]. However, excessive ROS accumulation, often triggered by genetic mutations (e.g., in TP53 or KEAP1) or environmental insults, can induce DNA damage, genomic instability, and epigenetic alterations, directly facilitating oncogenesis [4]. Significantly, ROS also drive TME remodeling through the activation of hypoxia-inducible factors (HIFs) and the promotion of angiogenesis, thereby creating a niche that supports tumor growth and immune evasion [6,7].

Inflammation, a physiological response to tissue injury or infection, becomes pathogenic when unresolved [8]. Chronic inflammation in the TME promotes tumorigenesis through the secretion of pro-inflammatory cytokines, such as tumor necrosis factor α (TNF-α), interleukin-6 (IL-6), and chemokines, which can recruit immune suppressive cells, such as regulatory T cells (Tregs) and myeloid-derived suppressor cells (MDSCs) [3,9]. These mediators not only suppress anti-tumor immunity but also directly drive malignant phenotypes by inducing epithelial–mesenchymal transition (EMT), metastasis, and metabolic reprogramming [10]. For instance, TNF-α and interleukin-1β (IL-1β) can enhance ROS production through NF-κB signaling, establishing a positive feedback loop that sustains both oxidative stress and inflammatory signaling [11].

The synergistic interplay between oxidative stress and inflammation further augments cancer’s aggressiveness [12]. On the one hand, DNA damage derived from ROS triggers the activation of inflammatory sensors, such as AIM2-like receptors (ALRs); on the other hand, inflammatory cytokines can amplify ROS production via nicotinamide adenine dinucleotide phosphate (NADPH) oxidases’ activation, thereby establishing a self-reinforcing cycle [11]. This intricate axis facilitates tumor cell survival, confers resistance to chemotherapeutic agents, and enables immune escape. For example, ROS-induced NF-κB activation enhances CXCL12 secretion, promoting metastasis to lymph nodes in a pancreatic tumor model [13]. In another report, in a colitis-associated colorectal cancer (CRC) model, chronic inflammation resulting in the upregulation of Cyclooxygenase-2 (COX-2) greatly elevates ROS levels and contributes to genomic instability [14].

Despite the increasing acknowledgment of these biological mechanisms, current therapies targeting either oxidative stress or inflammation alone often fail to achieve sustained therapeutic responses [3,15]. Antioxidants like N-acetylcysteine (NAC) may paradoxically promote tumor growth by scavenging ROS-dependent apoptotic signals [16]. Similarly, anti-inflammatory agents, such as aspirin or COX-2 inhibitors, struggle to address the TME’s complexity, often suppressing beneficial immune responses [17,18]. A paradigm shift is needed toward context-dependent therapeutic strategies that exploit the oxidative–inflammatory axis. Recent advances highlight the potential of dual-targeting approaches. For instance, combining Nrf2 activators (e.g., sulforaphane) with immune checkpoint inhibitors enhances T cell infiltration in hepatocellular carcinoma [19,20]. Pharmacological inhibition of NOX enzymes or KEAP1-NRF2 pathway regulators disrupts the oxidative stress–inflammatory feedback loop, sensitizing tumors to chemotherapy [21,22].

This review emphasizes current knowledge regarding the oxidative stress–inflammation axis, emphasizing its dual regulatory roles throughout tumor initiation, progression, and therapy resistance. We discuss mechanistic insights into their crosstalk, highlight diagnostic and prognostic biomarkers, and critically assess therapeutic strategies aimed at breaking the pro-tumorigenic cycle. Future studies should focus on designing precise medicines based on patient-specific biomarkers given the heterogeneity of tumors. Ultimately, targeting this dual axis offers a transformative opportunity to overcome therapeutic limitations and improve patient outcomes.

## 2. The Complex and Dual Relationships Between Oxidative Stress and Cancer

**Figure 1:**
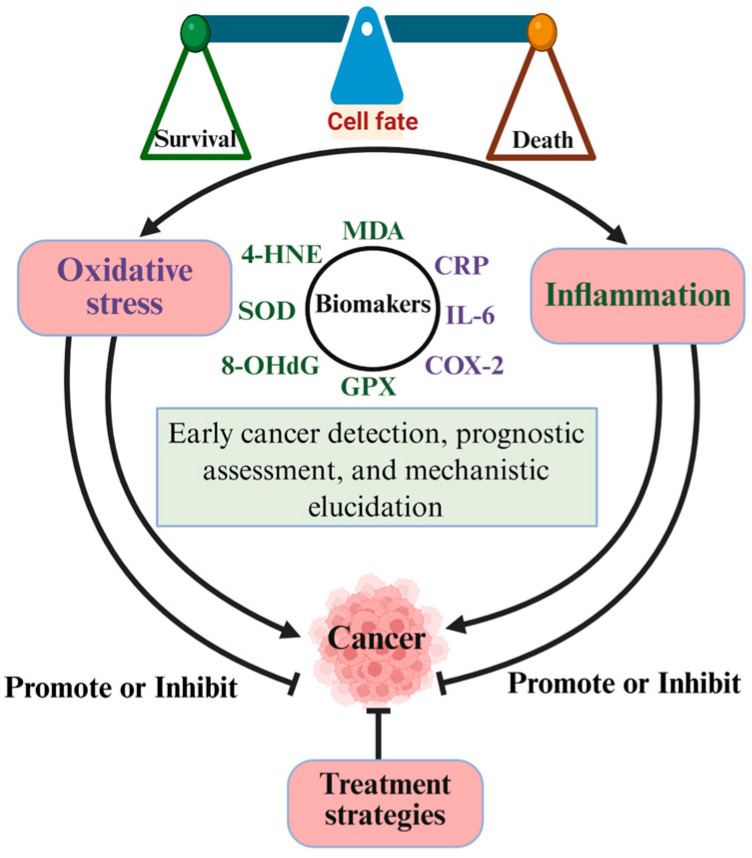
The complex relationship between oxidative stress and inflammation in cancer. Oxidative stress and inflammation mutually promote each other. They promote or inhibit the occurrence and development of cancer by regulating the levels of different biomarkers. Created with BioRender.com.

The complex relationship between oxidative stress and inflammation in cancer is shown in Figure 1. Oxidative stress mainly refers to the imbalance between the production of ROS/RNS, including superoxide anion (O_2_•^−^), hydroxyl radical (OH•), nitrogen dioxide (NO_2_•), alkoxyl/alkyl peroxyl (RO•/ROO•), hydrogen peroxide (H_2_O_2_), nitric oxide (NO), hypochlorous acid (HOCl), etc. [4,23,24], and the endogenous antioxidant defense system in the body, leading to a pathological state of oxidative damage [25]. The disruption of redox homeostasis has been associated with a wide range of human diseases, such as cancer, in which elevated levels of ROS are thought to play a pivotal role in the development and progression of tumors [7,25,26,27]. Researchers speculate that tumor tissues generate relatively higher levels of ROS due to metabolic changes, inflammation, hypoxic conditions, and the upregulation of ROS-generating enzymes driven by oncogenes [26,28]. Elevated ROS levels can lead to DNA mutations and facilitate oncogenic signaling pathways, thereby propelling tumor initiation and progression. However, excessively high ROS levels can also induce tumor cell death [29,30], suggesting that cancer prevention could potentially be achieved by reducing these relatively high ROS levels. In this section, we comprehensively discuss the dual effects of oxidative stress on promoting and inhibiting tumorigenesis and development.

### 2.1. The Generation of ROS

**Figure 2:**
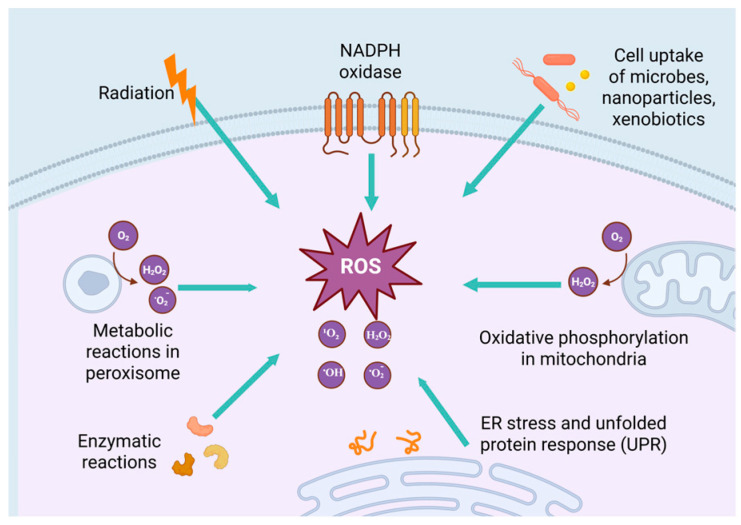
Elevated ROS levels are associated with many factors. In the process of mitochondrial oxidative phosphorylation, when the electron transport chain (such as complex I and III) is dysfunctional, electrons easily combine with oxygen to form O_2_•^−^, which in turn converts to H_2_O_2_ and other ROS. NADPH oxidase is a transmembrane enzyme complex. Its core function is to catalyze the transfer of electrons from NADPH to oxygen to generate O_2_•^−^, which is further converted to H_2_O_2_, OH•, and other ROS. Cellular uptake of microorganisms, nanoparticles, and xenobiotics is an important physiological process for organisms to cope with external stimuli. However, the intracellular responses triggered by the uptake of microorganisms, nanoparticles, and xenobiotics are often accompanied by ROS generation and oxidative stress. Radiation can directly damage DNA, lipids, and proteins and induce cells to produce ROS. Peroxisomes play a key role in maintaining cellular REDOX homeostasis by catalyzing the generation and scavenging of ROS, the balance of oxidative metabolic pathways, and the interaction with other organelles. Enzymatic reactions generate ROS (such as ^1^O_2_, O_2_•^−^, H_2_O_2_ and OH•) through catalytic processes, disrupting cellular redox balance and leading to oxidative stress. ER stress and unfolded protein response interfering with steady-state, metabolic balance, and intracellular calcium signaling pathways ultimately lead to ROS excess generation. Created with BioRender.com.

ROS are highly reactive free radicals, ions, or molecules characterized by a single unpaired electron, including species like ^1^O_2_, O_2_•^−^, H_2_O_2_, and OH• [31]. As pivotal signaling molecules, the regulation of ROS levels and redox homeostasis is critical for determining cellular functions and cell fate [32]. In cancer cells, elevated ROS levels can arise from diverse signaling pathways (Figure 2), including the activation of oncogenes or tumor suppressor genes, self-sustaining growth factor signaling, chronic inflammatory microenvironments, exposure to radiotherapy and chemotherapy, increased metabolic demands, and mitochondrial dysfunction [7,33,34].

Mitochondrial-derived ROS in cancer cells. Cancer cells utilize glucose through aerobic glycolysis (the Warburg effect) while maintaining mitochondrial activity [35]. Mutations in mitochondrial DNA and genes associated with the electron transport chain are commonly observed in tumors, leading to improper assembly of respiratory chain complexes and subsequent electron leakage [36,37]. It is widely acknowledged that mitochondrial ROS are primarily generated by Complexes I and III of the electron transport chain as superoxide within the mitochondrial matrix. Superoxide dismutases (SODs) present in the cytoplasm and mitochondrial matrix can convert O_2_•^−^ into O_2_ and H_2_O_2_, which regulate diverse cellular signaling pathways.

Growth factor and inflammatory cytokine signaling derived ROS in tumor cells. Self-sufficiency in growth signal transduction and chronic inflammation are two key hallmarks of tumor progression [38]. Signaling from growth factors and inflammatory cytokines increases the activity or expression of NOXs, membrane-bound enzyme complexes that generate O_2_•^−^ using electrons from NADPH [39]. Growth factors and cytokines, such as platelet-derived growth factor (PDGF), epidermal growth factor (EGF), transforming growth factor β (TGFβ), and TNFα, promote ROS production by activating NOXs. In the TME, elevated ROS levels originate not only from tumor cells but also from tumor-infiltrating immune cells (e.g., neutrophils and macrophages) [40]. For example, macrophage-derived NOX1 expression enhances inflammation and hepatocarcinogenesis in mouse models [41]. Thus, targeting NOXs to mitigate ROS-dependent pro-tumorigenic signaling has been proposed as a promising anticancer strategy.

ROS associated with oncogenic transformation. Oncogenic transformation promotes oxidative stress by remodeling cellular metabolism and enhancing mitochondrial ROS production [41]. The oncogenic activation of K-Ras in colon cancer cells redirects cellular metabolism towards aerobic glycolysis in the cytoplasm and enhances the activity of tricarboxylic acid (TCA) cycle activity within mitochondria [41,42]. Sustained energy generation via the TCA cycle generates NADH/FADH_2_, which amplifies electron flow through the electron transport chain and ROS generation, thereby facilitating tumorigenesis. Nevertheless, in normal human fibroblasts, the enhanced expression of c-Myc and H-RasV12 induces oxidative stress while simultaneously reducing mitochondrial ETC activity, resulting in non-mitochondrial superoxide [43], indicating that the origin of oncogene-induced oxidative stress may depend on the cellular context. Oncogenic signaling can also differentially regulate the expression of ETC subunits, giving rise to imbalanced ETC complex assembly and unstable electron transfer [43]. Significantly, mutations in mitochondrial-encoded ETC genes caused by ROS-induced mitochondrial DNA damage further disrupt mitochondrial redox homeostasis and promote cancer progression [44].

### 2.2. The Mechanism of ROS Based on Oxidative Stress Promoting Tumor Development

Oxidative stress drives tumorigenesis. Genomic instability, coupled with mutational accumulation, represents a fundamental characteristic of carcinogenesis [45]. This variability is caused by internal factors, such as the spontaneous accumulation of mutations and external factors (e.g., the environment and radiation) [46]. ROS, one of the common cellular mediators for both intrinsic and extrinsic carcinogenic factors, can directly impair nuclear DNA, resulting in genomic instability, such as single/double-strand breaks, point mutations, or chromosomal breaks, which subsequently activate the expression of proto-oncogenes (such as RAS and MYC) or inactivate tumor suppressor genes (such as TP53 and PTEN), along with DNA damage-repair-related genes, thereby driving tumorigenesis [47,48]. Moreover, ROS induces mutagenic lesions by damaging DNA bases (e.g., the oxidation of guanine to 8-oxo-dG) or inhibiting repair enzymes (e.g., ATM, BRCA1), thus triggering the repair of mutagenic lesions and SOS response, a stress-induced DNA damage response. This halts replication forks and promotes a transition of DNA polymerases from high-fidelity to error-prone forms, leading to additional oncogenic mutations [49,50]. Research has demonstrated that ROS can oxidize cysteine residues to activate the three most prevalent oncogenic switch genes (HRAS, NRAS, and KRAS) in human cancers, ultimately influencing tumorigenesis [51]. In a Kras-mediated lung cancer mouse model, mitochondrial ROS promotes tumor development via the ERK-MAPK signaling pathway [42]. In Kras-driven pancreatic cancer mouse models, the development and progression of premalignant lesions were significantly suppressed upon inhibition of ROS using NAC and MitQ [52]. Epigenetic regulation is another critical mechanism associated with the expression of tumor-associated genes [53]. ROS has been identified as a catalyst for DNA methylation modifications, upregulating DNA methyltransferase (DNMT) expression or forming DNMT-containing complexes. These processes extensively participate in the regulation of aberrant hypermethylation of tumor suppressor gene (TSG) promoters (e.g., CDX1, which governs intestinal epithelial proliferation and differentiation) and global hypomethylation, thereby downregulating TSG expression and promoting tumorigenesis [54,55,56]. In summary, oxidative-stress-driven DNA damage, mutations, or epigenetic modifications (including methylation and histone modifications) alter gene expression, acting as key determinants of tumor initiation, angiogenesis, metastasis, and treatment resistance (Figure 2).

Oxidative stress promotes tumor cell proliferation. Oxidative stress, mediated by ROS, directly influences the characteristic features of malignant tumors, including uncontrolled proliferation and growth [57,58]. For example, ROS induced by 5-lipoxygenase (5-LO) and NADPH oxidase 4 (NOX4) significantly augment pancreatic cancer cell survival [59]. The mechanisms of oxidative-stress-promoting tumor growth are multifarious. For instance, ROS generated under oxidative stress can stimulate tumor proliferation by activating pro-survival signaling pathways. Researchers have observed that administering the antioxidant NAC to mice bearing tumor xenografts decelerated tumor growth, which was attributed to reduced HIF-1α expression, demonstrating the critical role of ROS in driving cancer progression [60]. Thus, oxidative signaling-activated HIF-1 enhances tumor survival and progression by upregulating genes that govern glycolysis, angiogenesis, and cellular metabolism. Simultaneously, ROS can activate the PI3K/Akt/mTOR and NF-κB pathways by modifying kinases like MAPK and PI3K/AKT, facilitating tumor cell proliferation and suppressing apoptosis [61]. Moreover, ROS function as a second messenger molecule to directly mediate the activation of growth factors, such as PDGF [62], EGF [63], and MAPK [62], or induce the inactivation of phosphatase and tensin homolog (PTEN), thereby promoting tumor cell proliferation [64]. For example, the copper chaperone for superoxide dismutase (CCS) promotes breast cancer cell proliferation via ROS-mediated MAPK/ERK signaling [65]. H_2_O_2_-induced PTEN inactivation hyperactivates the PI3K/AKT/mTOR pathway, further facilitating breast cancer cell proliferation [66].

Oxidative stress promotes and maintains tumor metastasis. Emerging evidence suggests that elevated ROS levels are essential for promoting and sustaining invasive behaviors in cancer cells [67]. In the early stages, tumor cells often utilize the EMT process to invade adjacent stromal tissue [68,69]. During this transition, ROS promotes tumor metastasis through multiple mechanisms [64]. First, ROS induces Rho family GTPase-dependent cytoskeletal reorganization. Rho family proteins govern the dynamic changes in the cytoskeleton, and ROS can enhance their activity to alter cell morphology and motility, facilitating tumor cell migration [64]. Second, ROS promote the degradation of the extracellular matrix (ECM) through protease-dependent mechanisms (e.g., via matrix metalloproteinases and serine proteases), thereby driving tumor cell metastasis [70,71]. As the ECM serves as a critical microenvironmental scaffold for cell survival, its degradation would disrupt the structural support, enabling tumor cells to breach basement membranes and infiltrate surrounding tissues, thereby enhancing invasive and metastatic phenotypes [64]. Emerging evidence highlights the critical role of ROS in tumor metastasis. For instance, in clear cell renal cell carcinoma (ccRCC), elevated ROS production activates the MAPK signaling pathway, leading to the overexpression of matrix metalloproteinase-2 (MMP2), a protease responsible for degrading ECM components. This overexpression enhances the invasiveness of ccRCC cells, driving the progression of renal cell carcinoma [72]. Conversely, aberrant expression of serine-threonine kinase A (AURKA) is observed in oral squamous cell carcinoma (OSCC). Knockdown of AURKA increases intracellular ROS levels, which, in turn, inhibits the EMT process, demonstrating that the regulatory mechanisms of ROS-interacting proteins on EMT vary among different tumor types, reflecting the complexity of oxidative stress in cancer progression. Furthermore, ROS accelerate HIF-1α-dependent angiogenesis, as tumor metastasis requires adequate blood supply, and ROS-activated HIF-1α promotes new blood vessel formation, thus providing pathways for distant tumor cell metastasis [64]. Additionally, elevated ROS levels in tumor cells activate HSF1, matrix metalloproteinases, and NF-κB, all of which collectively promote metastasis [72,73,74]. For example, ROS-induced activation of NF-κB can downregulate epithelial cadherin (E-cadherin) to disrupt intercellular adhesion while upregulating N-cadherin and vimentin, thereby triggering EMT and driving tumor cell metastasis. Moreover, ROS, a signaling molecule, can activate the ERK signaling pathway by downregulating DUSP6 expression, further enhancing tumor invasiveness and metastasis [75].

ROS facilitate tumor multidrug resistance. In cancer therapy, chemotherapy drugs and radiotherapy rely on inducing excessive ROS production within cancer cells as a fundamental mechanism to eliminate them. However, many cancer cells gradually develop resistance to chemotherapeutic agents after treatment, significantly limiting therapeutic efficacy [76,77,78]. Research suggests that ROS-dependent drug resistance may arise from the activation of ROS-negative feedback regulatory systems, including HIF-1α, glutathione (GSH), xCT, thioredoxin reductase (TrxR), Nrf2, NADPH/NADP+, MnSOD, and catalase [78]. For example, Nrf2 is a key transcription factor for antioxidant defense. Under normal conditions, Nrf2 remains inactive and bound to Kelch-like ECH-associated protein 1 (Keap1). Upon elevation of intracellular ROS levels, ROS can oxidize cysteine residues on Keap1, causing Nrf2 dissociation. Free Nrf2 then translocates to the nucleus, binds to antioxidant response elements (AREs), and activates the transcription of antioxidant genes, such as glutathione S-transferase (GST) and heme oxygenase-1 (HO-1). These antioxidant proteins enhance cellular antioxidant defenses, alleviating chemotherapy-induced oxidative damage and, consequently, endowing cancer cells with drug resistance. Recent studies have revealed that chemotherapy-induced excessive ROS are sensed by the transcription factor aryl hydrocarbon receptor (AhR). The free sulfhydryl group of cysteine at position 300 (C300) in AhR undergoes sulfenylation (SH → S-OH), causing the modified AhR to dissociate from the HSP90 complex and be recruited to glycogen-targeting protein (PTG). By competing for the PP1 phosphatase binding site, the modified AhR inhibits the dephosphorylation of glycogen phosphorylase liver form (PYGL), thereby promoting glycogen breakdown. This process redirects glucose-6-phosphate (G6P) derived from glycogenolysis into the pentose phosphate pathway (PPP), generating NADPH to eliminate ROS produced by the CYP450-mediated metabolism of chemotherapeutic agents. This mechanism allows tumor cells to evade oxidative-stress-induced lethality and drive drug resistance in cancer patients [77]. Furthermore, ROS can activate signaling pathways like PI3K/AKT and AMPK, which enhance tumor cell survival under chemotherapeutic stress [79]. For instance, ROS-induced PI3K/Akt signaling and abnormal nuclear activation of β-catenin can promote HIF-1α overexpression, conferring resistance to 5-fluorouracil (5-FU) in colorectal cancer cells [79]. These findings highlight the complex interplay between ROS signaling and cellular adaptive mechanisms in chemotherapy resistance. ROS may also stimulate cancer stem cell differentiation [80], promote EMT [81], and induce metabolic reprogramming [82], all of which contribute to the increased chemoresistance of cancer cells. The overexpression of ATP-binding cassette (ABC) transporter family members is a key mechanism underlying tumor drug resistance. ROS may upregulate ABC transporter expression, enabling these proteins to utilize the energy derived from ATP hydrolysis to efflux chemotherapeutic agents from cells, thereby reducing intracellular drug concentrations and conferring resistance [78]. Additionally, ROS promote the development of multidrug resistance (MDR) by modulating intracellular metabolic pathways. For instance, ROS-induced metabolic reprogramming shifts cancer cells towards aerobic glycolysis, providing rapid energy and biosynthetic precursors for tumor growth while enhancing tolerance to chemotherapeutic agents. Simultaneously, ROS influence the autophagy of cancer cells [83]. Autophagy, a cellular self-degradation mechanism, helps cancer cells in eliminating damaged organelles and proteins to maintain homeostasis. ROS activates autophagy-related signaling pathways, promoting autophagy, which enables cancer cells to survive chemotherapeutic stress, thereby establishing drug resistance [84,85].

### 2.3. Mechanisms of ROS Based on Oxidative Stress Impeding Tumor Development

**Figure 3:**
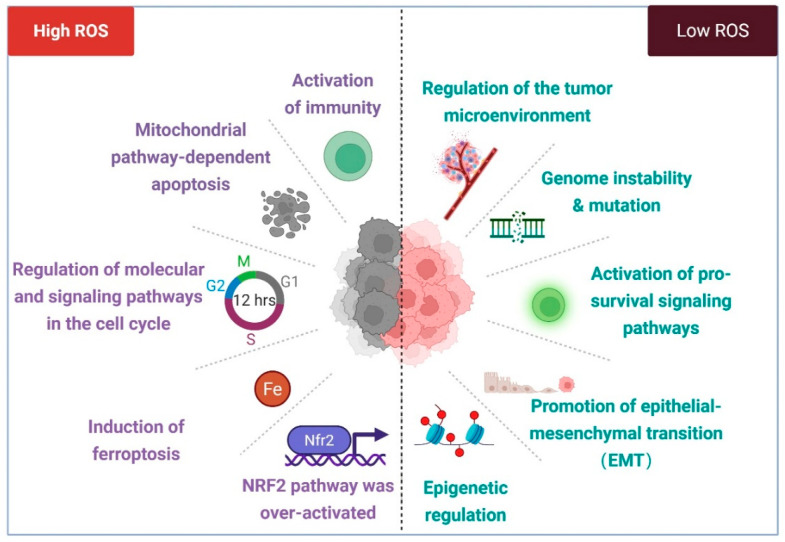
ROS in cancer biology. Excessive levels of ROS can inhibit tumor growth by triggering apoptosis, ferroptosis, or cell cycle arrest and even activate immune cells to recognize abnormal cells. In addition, it can inhibit the occurrence and development of tumors by activating Nrf2. However, relatively low levels of ROS can lead to genomic instability or mutation, thereby inducing the activation of proto-oncogenes or the inactivation of tumor suppressor genes and promoting the transformation of normal cells into precancerous lesions. At the same time, low levels of ROS can also promote the occurrence and development of cancer by activating pro-survival signaling pathways, promoting epithelial–mesenchymal transition and regulating epigenetics. Created with BioRender.com.

Under normal physiological conditions, high ROS levels during oxidative stress can inhibit tumor cell proliferation or promote tumor cell death through multiple mechanisms [30]. Excessively high ROS levels can induce diverse forms of tumor cell death (Figure 3). In the mitochondrial apoptotic pathway triggered by excessive ROS, oxidative stress disrupts the permeability of the mitochondrial inner membrane and mitochondrial membrane potential, activating signaling pathways like Bax/Bcl-2-cyt c, p38 MAPK, and phosphorylated HSP27. This leads to the release of cytochrome c and activation of the caspase family, ultimately driving the apoptotic death of cancer cells [86,87,88,89]. Moreover, studies have demonstrated that ROS can disrupt calcium homeostasis in the endoplasmic reticulum (ER) by impairing the gating functions of ryanodine receptors (RyR) and inositol 1,4,5-trisphosphate receptors (IP3R) while simultaneously inhibiting Ca^2+^-ATPase activity, thereby inducing cancer cell apoptosis [90,91]. Recent research has further highlighted that the lethal accumulation of lipid ROS in multiple tumor types triggers ferroptosis, an iron-dependent form of cell death [92,93]. Elevated iron concentrations and ROS levels stimulate the expression of tumor suppressor p14^ARF^ (CDKN2A), activate p53, and inhibit NRF2 activity, which collectively promote ferroptosis [94]. Under oxidative stress conditions, high levels of ROS can also suppress tumor cell proliferation by inhibiting proliferation-associated signaling pathways. For instance, excessive ROS inhibits AKT-dependent signaling pathways, thereby restraining proliferation in colorectal cancer cells [95]. Additionally, the persistent activation of cell cycle inhibitors by ROS can inhibit tumor growth [96,97]. The most commonly reported inhibitors are p21 (CDKN1A) and p16 (CDKN2A). Their accumulation leads to hypophosphorylation of the retinoblastoma protein (RB), which can inhibit the transactivation of E2F genes involved in nucleotide metabolism and DNA synthesis [98]. This results in cell cycle arrest and ultimately suppresses cell proliferation [99]. A notable phenomenon under oxidative stress is the translocation of p53 to the mitochondria, where it regulates mitochondrial membrane potential and triggers apoptosis [100,101]. Furthermore, studies have demonstrated that several proteins interacting with p53 are localized to the mitochondria, including MDM2, FOXO3a, and Parkin [102,103,104].

In addition to directly killing tumor cells, ROS can indirectly inhibit tumor progression through mechanisms like enhancing therapeutic sensitivity, modulating immune responses [105], and suppressing tumor stem-like properties. For instance, radiotherapy generates massive ROS, which collaborate with chemotherapeutic agents like cisplatin, doxorubicin, and oxaliplatin to induce DNA crosslinking damage, ultimately leading to cancer cell death [106,107,108,109]. Additionally, acute oxidative-stress-induced ROS promote the release of tumor antigens [110], activate dendritic cells (DCs) and CD8+ T cells [105,111], and enhance the efficacy of immune checkpoint inhibitors [112]. Furthermore, oxidative stress can suppress tumor stem-like characteristics, thereby affecting tumor cell survival [113,114,115].

### 2.4. Spatiotemporal Regulation of ROS Under Oxidative Stress

ROS play a complex and dynamic dual role in tumorigenesis and tumor progression, exhibiting both pro-tumorigenic and anti-tumorigenic activities, a long-standing paradox in the field. Discrepancies and even contradictory findings reported by different researchers have ignited controversy regarding the role of antioxidants in cancer therapy. These discrepancies likely stem from differences in experimental models and methodologies [75]. A recent study demonstrated that ROS exhibit distinct functions at different stages of pancreatic cancer development; they suppress pre-neoplastic lesions yet promote metastasis in pancreatic cancer. This dynamic regulatory mechanism may be attributed to the pivotal regulatory factor TIGAR [75]. The spatiotemporal regulation of ROS has become a critical focus in tumor biology research, underscoring the necessity to elucidate the context-dependent mechanisms that govern their dual roles in cancer.

From a temporal perspective, oxidative stress levels exhibit dynamic changes throughout tumor progression. During the initiation phase of tumorigenesis, localized elevation of ROS facilitates the transformation of normal cells into malignant phenotypes by inducing DNA oxidative damage and genomic instability, thereby activating oncogenes and inactivating tumor suppressor genes [25,29,75,116,117]. Simultaneously, ROS activate redox-sensitive survival pathways, such as NF-κB, HIF-1α, and Nrf2, through spatiotemporal dynamic fluctuations to remodel the TME to enhance the release of inflammatory cytokines, thus supporting the survival and proliferation of tumor cells [25,29,75,116,117]. As tumors advance to advanced stages, the continuous accumulation of ROS may surpass cellular antioxidant defenses, leading to genomic instability and cell death. Paradoxically, ROS may also promote the acquisition of invasive and metastatic phenotypes [7,26,33,118,119,120,121]. Notably, therapeutic interventions significantly alter redox kinetics; radiotherapy, many chemotherapeutic agents, and photodynamic therapy exert their anti-tumor effects by further increasing ROS levels [78,122,123,124,125,126,127]. Nevertheless, cancer cells may develop resistance by upregulating antioxidant defense systems, such as the glutathione system and the thioredoxin system, to counteract ROS-induced cytotoxicity [27,118,128,129,130,131,132,133].

From a spatial perspective, oxidative stress levels differ significantly across distinct tumor regions [83,134,135,136]. The tumor core region typically exhibits chronic hypoxia due to the abnormal vasculature and insufficient blood perfusion, leading to mitochondrial electron transport chain dysfunction. This results in increased ROS production, accompanied by reduced ROS scavenging capacity, thereby facilitating the emergence of invasive phenotypes [26,83]. For example, after cisplatin treatment, the core region shows a marked elevation in Z-RNA levels, triggering necroptosis, while the margin region may demonstrate a weaker response due to non-uniform drug distribution or microenvironmental barriers [137]. Conversely, the tumor margin region features relatively abundant vasculature and elevated oxygen tension but undergoes intermittent hypoxia–reoxygenation cycles, further amplifying oxidative stress fluctuations. In glioblastoma (GBM), tumor-associated microglia undergo immunosuppressive transformation under oxidative stress, resulting in reduced antigen-presenting capacity. This phenomenon is more pronounced in the tumor’s core, whereas the margin region may exhibit lower oxidative stress because of differences in vascularization and immune cell infiltration [135]. Moreover, variations in extracellular matrix density and GSH levels within the tumor influence the distribution of oxidative stress. Nanoparticles, for instance, can deplete GSH in the core region and catalyze H_2_O_2_ to generate hydroxyl radicals, thereby intensifying oxidative stress in the core. However, the margin region may exhibit weaker responses due to differences in drug permeability [134]. At the subcellular level, mitochondria, the ER, peroxisomes, and plasma membranes are primary sites of ROS generation. ROS produced by different organelles exhibit distinct chemical properties and biological effects. For example, mitochondria predominantly generate O_2_•^−^, while the ER generates H_2_O_2_ during protein-folding processes.

Clinical assays demonstrate that in patients with high-grade serous ovarian cancer (HGSOC), downregulation of VPS35 in hypoxic regions leads to mitochondrial translation inhibition, contributing to resistance against multiple ROS-dependent anticancer drugs [83]. Additionally, during pre-invasive stages, tumor cells enhance local ROS clearance by upregulating antioxidant enzymes like SOD2 and GPX4, which aid in evading immune surveillance and enhance metastatic potential [26,117]. These findings suggest that the spatiotemporal regulation of ROS and its dynamic effects throughout tumor progression stages may partially elucidate the controversial roles of antioxidants in cancer therapy. Understanding the spatiotemporal dynamics of ROS balance in tumors could guide the formulation of stage-specific therapeutic strategies. For instance, antioxidants might be employed in early preventive interventions, while pro-oxidant agents engineered to circumvent tumor antioxidant defenses could be applied in late-stage metastatic phases. Such approaches could exploit the context-dependent roles of ROS to optimize therapeutic efficacy.

## 3. Inflammation in Cancer

### 3.1. The Link Between Inflammation and Cancer

Since its discovery, inflammation has emerged as a research focus due to its significant role in regulating various diseases, particularly in cancer biology [138,139]. Over the past decades, numerous studies have extensively explored the influence of inflammation on tumor initiation, invasion, metastasis, and drug resistance. Among the crucial factors contributing to tumorigenesis, the role of inflammation, especially chronic inflammation, has been the subject of extensive investigation. Although inflammation and cancer manifest as separate pathological conditions, they are, in fact, closely intertwined. Inflammation, an inherent defense mechanism, is triggered by tissue injury or pathogen invasion, aiming to facilitate repair and eliminate harmful entities. However, when inflammation becomes persistent and progresses to a chronic state, it gives rise to a permissive microenvironment that promotes carcinogenesis [138].

**Figure 4:**
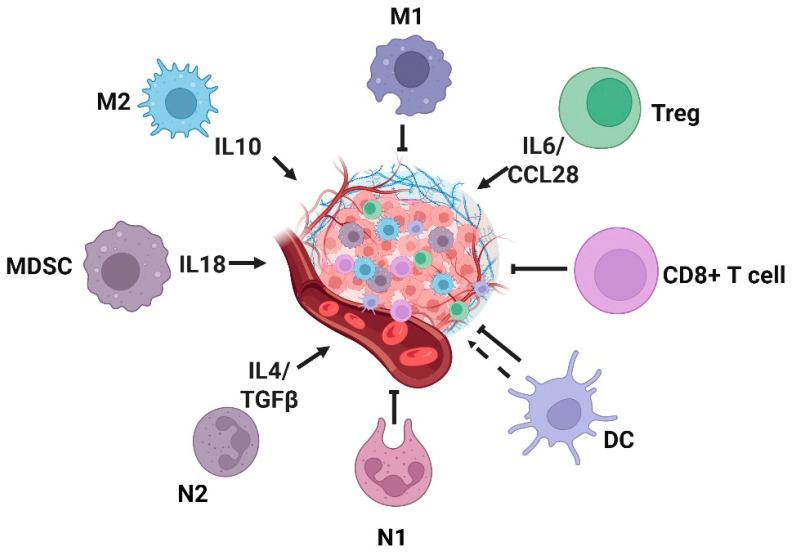
Tumor microenvironment and inflammatory cells. Inflammatory cells and inflammatory factors are involved in the development of tumors. M2, MDSC, N2, and Treg cells promote the occurrence and development of tumors in the tumor microenvironment, while M1, CD8+T, and N1 cells inhibit the occurrence and development of tumors. M1: M1-type macrophages; M2: M2-type macrophages; MDSC: myeloid-derived suppressor cells; N1: N1-type neutrophils; N2: N2-type neutrophils; DC: dendritic cell. Created with BioRender.com.

Chronic inflammation and tumor progression are intricately linked via the TME, which is shaped by inflammatory mediators, including inflammatory cells and cytokines. Inflammatory cells, such as macrophages and lymphocytes, accumulate and continuously secrete pro-inflammatory cytokines like TNF-α and IL-6 (Figure 4). On the one hand, these cytokines can stimulate genetic mutations in normal cells and disrupt normal intracellular signaling pathways, such as activating the NF-κB pathway, which dysregulates the balance of meticulously controlled cell proliferation and apoptosis processes, ultimately leading to abnormal cell proliferation. On the other hand, these cytokines also promote tumor angiogenesis, continuously supplying nutrients to cancer cells and facilitating their growth and metastasis. A well-documented example is the elevated risk of CRC associated with ulcerative colitis and Crohn’s disease [140,141]. Moreover, infections with *Helicobacter pylori*, hepatitis B, and hepatitis C viruses are also associated with increased cancer risk [142,143]. The recurrent tissue damage and repair caused by chronic inflammation drive continuous cellular proliferation. During this process, the probability of DNA replication errors increases, and mutations in pivotal tumor suppressor genes or oncogenes can ultimately propel normal cells towards malignant transformation.

From a mechanistic perspective, chronic inflammation drives carcinogenesis through diverse pathways. For example, in chronic inflammation induced by microbial infections, immune cells, such as macrophages at inflammatory sites, can produce ROS, leading to sustained DNA damage and subsequent mutations [144]. Moreover, cytokines secreted by immune cells, including TNF-α and macrophage migration inhibitory factor (MIF), can inhibit the activation of p53 and the Rb-E2F pathway, thereby facilitating tumorigenesis [145]. Components of the inflammatory process form self-reinforcing positive feedback loops that support cancer progression. Inflammatory and growth factors further stimulate transcription factors, such as NF-κB, collectively promoting the formation of an inflammatory TME [146,147]. The inflammatory TME is highly dynamic and complex, consisting of cellular components, such as tumor-associated macrophages (TAMs), tumor-associated neutrophils (TANs), DCs, MDSCs, and T lymphocytes [147]. These tumor-infiltrating cells collectively maintain an inflammatory milieu that enables tumor growth and induces immune suppression during tumor progression.

### 3.2. Role of Key Inflammatory Cells in Tumorigenesis and Progression

Inflammatory cells serve as a critical mediator of the body’s immune response, playing pivotal roles in recognizing and phagocytosing pathogens, as well as clearing damaged tissues to combat infections and promote tissue repair. Within the TME, distinct populations of inflammatory cells exert diverse effects on tumorigenesis and progression. Neutrophils, the principal effector cells during inflammatory responses and the most abundant white blood cells, are categorized into N1 and N2 subtypes. N1 neutrophils exhibit anti-tumor activity, whereas N2 neutrophils promote tumor growth [148]. Notably, TANs in the TME predominantly display an N2 phenotype and drive tumor metastasis through multiple mechanisms. Studies have demonstrated that TANs may promote tumor angiogenesis by inducing sustained release of vascular endothelial growth factor (VEGF) from peripheral endothelial cells or by secreting pro-inflammatory and immunosuppressive factors, such as IL-1β, IL-17, TNF-α, CCL4, MMP-9, CXCL8, and ANG1 [149]. Tumor-derived cytokines IFN-γ and GM-CSF drive TANs’ polarization by upregulating the expression of specific neutrophil activation markers, thereby enhancing anti-tumor activity [150]. Additionally, tumor-secreted TGF-β promotes the paracrine production of CCL2 and CCL17, recruiting N2-polarized neutrophils and establishing an immunosuppressive microenvironment [151,152]. Moreover, tumor cells induce tumor-associated neutrophils to release neutrophil extracellular traps (NETs) through autocrine signaling via IL-8/CXCL8, CXCR1/CXCR2 agonists, G-CSF, and TGF-β pathways. NETs can promote metastasis by inducing EMT [153,154,155,156,157,158,159]. However, the functional roles of NETs released by tumor-associated neutrophils are not fixed, and they are contingent on the TME context. For instance, in vitro-generated NETs have been shown to inhibit tumor growth in CRC and head and neck squamous cell carcinoma (HNSCC) by inducing apoptosis and suppressing proliferation [160,161,162]. Furthermore, co-culture experiments with melanoma cells revealed that NETs induced necrosis in melanoma cells [162]. These findings highlight the biphasic regulation of tumor-associated neutrophils in tumorigenesis and progression, with their effects determined by the dynamic state of the TME.

Similarly to tumor-associated neutrophils, TAMs can be classified into distinct subtypes, including M1-type macrophages and M2-type macrophages [163], with their polarization determined by molecular cues within the TME. Tumor cells use the plasticity of macrophages to secure their survival. In the early stages of tumor development, macrophages polarize towards the M1 phenotype, showing anti-tumor responses; however, in advanced stages, they shift towards the M2 phenotype to promote tumor progression [164]. This shift in polarization probably reflects variations in the molecular composition of TME across different phases of tumor development. Studies demonstrate that cytokines, such as IL-4, M-CSF/CSF1, IL-10, IL-33, IL-21, and TGF-β, drive TAMs towards the M2 phenotype, whereas M1-type TAMs are activated by TNF-α or GM-CSF [138]. M1 macrophages exert anti-tumor effects through multiple mechanisms, including inducing direct cytotoxicity via the secretion of ROS and NO [165] and mediating antibody-dependent cell-mediated cytotoxicity (ADCC) [166]. Conversely, M2 macrophages predominantly facilitate tumor progression by activating endothelial cell responses to growth factor signaling via CXCL12, IL-1β, IL-8, and Sema4d, thereby leading to the upregulation of angiogenesis-related genes and enhanced vascularization [167,168,169]. Additionally, M2 TAMs assist in tumor invasion and metastasis by indirectly degrading the tumor extracellular matrix [170].

DCs, originating from the bone marrow, are indispensable inflammatory cells that exert significant functions within inflammatory TMEs. In the context of the TME, tumor-infiltrating DCs exhibit compromised capacity to present tumor antigens, leading to T cell tolerance and the loss of anti-tumor immune function [171]. Moreover, cytokines secreted by tumor cells can modulate the maturation state of DCs, promoting pro-tumorigenic inflammatory responses. For instance, tumor-secreted IL-6 and M-CSF can transform mature DCs into macrophages and impede the priming of tumor-specific T cells [172]. Additionally, the expression of PD-L1 and PD-L2 on the surface of DC may inhibit the production of cytokines required for T cell activation [173].

MDSCs have been extensively characterized in the context of inflammatory tumors. Research has shown that MDSCs suppress anti-tumor immunity by either directly interacting with T cells or remodeling the TME through immunosuppressive cellular and molecular networks [138]. Upon being recruited to inflammatory tumor tissues in response to chemokines like CCL2, CCL5, CXCL8, and CXCL12, MDSCs rapidly produce immunosuppressive mediators, including ARG1, NO, TGF-β, and IL-10, which further impair anti-tumor immunity [174,175]. Additionally, tumor-derived factors, such as VEGF, IL-6, and IL-10, can recruit MDSCs to the inflammatory TME, where they activate the STAT3 signaling pathway to produce more VEGF, establishing a positive feedback loop that promotes tumor angiogenesis [176,177].

Additionally, regulatory T cells (Tregs) significantly drive tumor initiation, progression, and metastasis by establishing an immunosuppressive tumor microenvironment, remodeling inflammatory networks, and intervening in metabolic homeostasis [178]. Tregs directly inhibit the activation and proliferation of CD8+ cytotoxic T cells and Th1 cells by highly expressing immune checkpoint molecules (such as CTLA-4, PD-1) and secreting suppressive cytokines (IL-10, TGF-β) [179]. They also induce dendritic cells (DCs) to differentiate into an immunologically incompetent phenotype and recruit myeloid-derived suppressor cells (MDSCs) and M2-type macrophages to the tumor microenvironment (TME), forming an “immune desert”-type microenvironment that allows tumor cells to escape immune surveillance [180,181,182]. In chronic-inflammation-associated tumors, Tregs weaken the ability of inflammation to eliminate abnormal cells by suppressing Th1/Th17 cell-mediated pro-inflammatory responses while secreting chemokines, such as CCL22, to establish a “pro-cancer inflammation-immunosuppression” positive feedback loop. For example, they cooperate with macrophages via the CXCL12–CXCR4 axis to promote angiogenesis [183]. At the metabolic level, Tregs degrade ATP into immunosuppressive adenosine by highly expressing CD39, preferentially uptake glucose and tryptophan, deprive effector T cells of energy sources, and deplete their function [184]. In addition, VEGF and TGF-β secreted by Tregs can directly promote tumor angiogenesis and EMT and enhance the invasion ability of tumor cells. In the therapeutic scenario, Tregs mediate chemotherapy, radiotherapy, and immunotherapy resistance by maintaining PD-L1 expression or recruiting MDSCs [185]. Clinical studies have shown that the infiltration level of FOXP3^+^ Tregs in tumor tissues is negatively correlated with the prognosis of patients. Immune intervention strategies targeting Tregs, such as the depletion of Tregs by anti-CD25 monoclonal antibody and the blockade of their function by CTLA-4 inhibitors, have become the key direction of tumor immunotherapy [186,187,188].

Vascular endothelial cells are now widely recognized as pivotal inflammatory cells involved in the regulation of inflammatory tumors. Beyond their conventional role as “gatekeepers,” these cells can be subverted by inflammatory or cancer signals to facilitate immune cell infiltration and tumor dissemination. As the crucial barrier separating blood from tissues, endothelial cells actively participate in inflammatory responses. Upon stimulation by inflammatory signals such as TNF-α and IL-1, vascular endothelial cells undergo activation, during which they release chemotactic signals (e.g., CXCL8, CXCL12, complement C5a, and platelet-activating factor) and upregulate surface adhesion molecules, thus facilitating the transmigration of leukocytes into tissues [189,190]. Nevertheless, chronic inflammation can weaken vascular wall integrity, paradoxically enabling easier leukocyte transmigration and exacerbating inflammation. This creates a conducive microenvironment for cancer metastasis. Tumor cells metastasize by breaching vascular barriers, a process similar to leukocyte migration. However, due to their larger size, cancer cells often become trapped within blood vessels [191]. To overcome this obstacle, they utilize adhesion proteins, such as E-selectin, to “roll” along the vascular endothelium, much like a rolling mechanism, to identify suitable exit sites [192,193,194,195,196,197].

### 3.3. Role of Key Inflammatory Cytokines in Tumorigenesis and Progression

Cytokines, a class of low-molecular-weight peptides or glycoproteins, play critical roles in TME signaling by mediating pro-inflammatory or anti-inflammatory responses. Research has evidenced that numerous inflammatory cytokines are closely associated with tumor initiation and progression [198]. Under the distinctive pathophysiological conditions of the TME, cancer-associated inflammatory cytokines, including TNF-α, TGF-β, IFN-I, IL-1, IL-6, and IL-10, often exhibit upregulated expression, thereby modulating the biological behaviors of tumor cells [199].

Interleukin-1 (IL-1), a key pro-inflammatory cytokine, is aberrantly upregulated in diverse cancer types and correlates with adverse clinical outcomes in patients [199]. This cytokine exhibits a dual role in tumor biology, mediating both pro-tumorigenic and context-dependent anti-tumor effects. Mechanistically, IL-1, secreted by tumor cells or immunosuppressive myeloid populations, such as MDSCs and Tregs, promotes oncogenesis through multiple pathways; it stimulates the production of pro-tumorigenic factors (e.g., IL-6, TNF-α) to drive autocrine/paracrine tumor growth [200,201], facilitates immune evasion by recruiting MDSCs to suppress effector T cell activity [202], and induces VEGF to promote tumor angiogenesis [203,204]. Conversely, under specific microenvironmental contexts, IL-1 can also activate anti-tumor immune pathways, such as inducing Th1-type immune responses and enhancing dendritic cell maturation [205]. Despite these pro-immunity functions, IL-1 exerts a predominantly pro-tumorigenic role in most cancer settings. It orchestrates an immunosuppressive TME by fostering the accumulation of regulatory immune cells (e.g., Tregs, MDSCs), inhibiting effector T cell cytotoxicity, and promoting the expression of immune checkpoint molecules. These effects collectively enable tumors to evade immune surveillance and develop resistance to immunotherapies like PD-1/PD-L1 blockade. Given this mechanistic duality, targeting IL-1β, the biologically active isoform primarily associated with pathological inflammation, has emerged as a promising adjunct to combination immunotherapy. Preclinical studies demonstrate that IL-1β inhibition synergizes with immune checkpoint inhibitors to enhance anti-tumor efficacy in murine models, likely by reversing TME-mediated immune suppression [206]. However, clinical translation must account for the context-dependent effects of IL-1, as its dual pro- and anti-tumor functions vary across cancer types and stages. Therapeutic strategies should therefore be meticulously tailored to specific malignancies, integrating molecular profiling of the TME to optimize benefits while mitigating off-target inflammatory risks.

IL-6, a pro-inflammatory cytokine produced by diverse cell types, including tumor cells and inflammatory cells, drives tumorigenesis and progression through multiple molecular pathways. Functioning as a “conspirator” in tumor development, IL-6 not only promotes the uncontrolled growth and drug resistance of tumors but also facilitates metastasis and colonization, ultimately making cancer more challenging to treat. Research has demonstrated that IL-6 expedites tumor cell proliferation by upregulating cyclins, downregulating the expression of the cyclin-dependent kinase inhibitor p21, and activating STAT3 protein. Meanwhile, IL-6 activates the Ras-ERK and PI3K-Akt signaling pathways to further accelerate tumor growth [207,208]. Furthermore, IL-6 promotes the expression of the anti-apoptotic protein Bcl-2 through STAT3 signaling, enabling tumor cells to evade chemotherapy-induced cytotoxicity [209,210]. Other pro-tumorigenic mechanisms of IL-6 also involve the suppression of tumor senescence [211,212], collaboration with other growth factors to amplify malignant behaviors [213], the induction of EMT, and the promotion of angiogenesis [214,215,216].

As a pivotal mediator of anti-inflammatory responses, IL-10 cytokine is primarily produced by tumor cells and leukocytes. IL-10 is a cytokine with a “Janus-faced” nature. As an anti-inflammatory molecule, it suppresses excessive inflammatory reactions, such as by reducing the release of pro-inflammatory cytokines of certain immune cells, thereby facilitating the maintenance of tissue homeostasis [217]. Nevertheless, IL-10 derived from either tumor or immune cells suppresses immune function by creating an “immunosuppressive shield” that allows tumors to evade immune attack [218]. Under certain contexts, IL-10 can activate immune cells and directly stimulate anti-tumor responses by increasing IFN-γ secretion [219]. This dual functionality likely arises from cancer-type-specific and immune-cell-state-dependent mechanisms. Consequently, its role must be evaluated in the context of the specific TME and patient conditions when formulating therapeutic strategies.

TNF-α, a pro-inflammatory cytokine produced by immune cells and tumor cells [220], exerts a double-edged sword role in cancer. At low concentrations, TNF-α acts as a tumor “conspirator”, promoting cancer cell proliferation, metastasis and angiogenesis, and chemoresistance [221], along with reducing the infiltration of CD8+ T cells into tumor tissues [222] and enhancing the function of immunosuppressive Treg cells through the TNF receptor 2 (TNFR2) [223]. However, at high concentrations, TNF-α may directly induce tumor cell death [224]. Nevertheless, the TME predominantly sustains low TNF-α concentrations, where its pro-tumorigenic effects dominate. Hence, targeting TNF-α or its receptors TNFR2 has emerged as a viable anticancer strategy. Some studies have demonstrated that blocking TNFR2 can inhibit ovarian cancer cell growth [225] and that the combination of TNF-α inhibition with immune checkpoint inhibitors could enhance therapeutic outcomes. Nevertheless, meticulous consideration of its dual nature is imperative to prevent interference with beneficial immune responses. In the majority of instances, TNF-α acts as an oncogenic ally, facilitating tumorigenesis, metastasis, and immune evasion.

The Janus-faced role of TGF-β in cancer evolves dynamically according to the disease stage [226,227,228,229,230]. Under physiological conditions and during early tumorigenesis, TGF-β acts as a tumor suppressor, operating much like a “brake” to inhibit tumor growth. As tumors advance, TGF-β undergoes a paradoxical role switch, altering its function to promote tumor proliferation, metastasis, angiogenesis, and immune evasion. Specifically, TGF-β induces EMT, enabling cancer cells to detach from primary lesions [231,232,233], and facilitates immune escape by suppressing anti-tumor immune responses [234]. These mechanisms collectively render cancer cells more resistant to therapeutic eradication [226,228,229,230].

Type I interferons (IFN-I), which function as versatile mediators, also exhibit complex and paradoxical roles in the context of cancer. As pro-inflammatory signals, IFN-I can promote tumor growth and metastasis via multiple mechanisms, including modulation of the TME [235], the regulation of cancer cell stemness [236], and the induction of EMT in endothelial cells [237]. In chronic inflammation, IFN-I establishes a tumor-supportive microenvironment conducive to growth and progression [235,238] and facilitates tumor cell immune evasion [236,239,240]. Additionally, evidence has shown that IFN-I can enhance cancer cell stemness via the regulation of the epigenetic regulator KDM1B [236]. However, the anti-tumor activities of IFN-I have also been well-documented, as studies have demonstrated that IFN-I exert their anticancer effects by negatively regulating the formation of premetastatic niches within the TME [241].

Consequently, the regulatory roles of various inflammatory cytokines in tumorigenesis are not one-dimensional, as the majority of inflammatory cytokines possess Janus-faced characteristics; they can either serve as allies in the fight against cancer or act as traitors in promoting tumor development. Their ultimate effects are contingent upon factors like cancer types, disease stage, and other elements within the TME. In the development of therapeutic strategies, it is critical to precisely evaluate their mechanistic roles to avoid “one-size-fits-all” approaches. The cornerstone of effective therapy lies in capitalizing on their context-dependent properties to devise targeted interventions that are customized to the specific circumstances of individual tumors.

## 4. Crosstalk Between Oxidative Stress and Inflammation in Cancer

As two critical biological processes, oxidative stress and inflammation not only exert individual influences on physiological states but also act synergistically under pathological conditions, co-regulating the initiation and progression of cancer [242]. Understanding their intricate interplay is fundamental to elucidating the mechanisms underlying tumorigenesis [243]. Oxidative stress refers to an imbalance between oxidative and antioxidant defense systems, resulting in elevated levels of ROS or RNS [242]. Inflammation, a protective response of the body to harmful stimuli, entails the activation of immune cells, the release of inflammatory mediators, and tissue repair processes [242]. However, dysregulated inflammation can precipitate the pathogenesis of numerous diseases. In cancers, oxidative stress and inflammation establish a vicious cycle, both directly and indirectly promoting tumor cell proliferation, migration, invasion, and metastasis. Moreover, they jointly shape the TME, creating a milieu conducive to tumor progression [243].

ROS have been demonstrated to influence both cancer and inflammation [244,245,246]. Therefore, targeting ROS in tumors may represent a potential cancer treatment strategy [181]. Oxidative stress prompts the overproduction of ROS or RNS within the body, which can be eliminated by the body’s endogenous antioxidant system under normal physiological circumstances [242]. However, when the body’s antioxidant system fails to maintain the balance between the production and clearance of ROS or RNS, the excessive accumulation of ROS or RNS can trigger inflammatory responses [242,247]. Prolonged exposure to oxidative stress is associated with many inflammation-related diseases, such as chronic inflammatory disorders, inflammatory bowel disease (IBD), and cancer [242]. Oxidative stress activates or enhances inflammatory responses through multiple pathways, thereby facilitating the occurrence and development of tumors [11,242,248]. Excessive ROS or RNS can cause damage upon cellular structures by destroying the membrane lipids [243]. Subsequently, the damaged cells release a cascade of damage-associated molecular patterns (DAMPs), which initiate innate immune responses and trigger inflammation [243]. Moreover, ROS can activate key signaling molecules, such as the NF-κB, PI3K/Akt and MAPK inflammatory signaling pathways, leading to the release of inflammatory cytokines and further exacerbating local inflammatory responses [249,250,251]. Notably, the activation of the transcription factor NF-κB plays a critical role in regulating inflammation, angiogenesis, adaptive metabolism, and treatment resistance [252,253], and is involved in multiple stages of cancer development. This transcription factor serves as both a trigger and a target for ROS, leading to the deterioration of the redox status in tumor tissues [254]. Additionally, NF-κB can induce the release of pro-inflammatory cytokines, promote the formation of an inflammatory tumor microenvironment, contribute to cancer progression, and facilitate tumorigenesis. This indicates that NF-κB acts as a crossroads between oxidative stress and inflammation in cancer. Therefore, targeting ROS production and the formation of the tumor inflammatory microenvironment mediated by NF-κB represents a promising cancer treatment strategy [255,256]. Based on the current research landscape, several bioactive natural products and small molecules have been tested for their anti-tumor effects due to their regulatory roles in the ROS/NF-κB signaling pathway [257,258,259,260]. Furthermore, growing evidence demonstrates that mitochondrial ROS play a key role in the tumor inflammatory microenvironment. Numerous studies have shown that oxidative stress impairs mitochondrial function [261,262], and the release of mitochondrial DNA can activate inflammasomes, thus promoting the secretion of inflammatory factors [263,264,265]. In the tumor microenvironment (TME), active oncogenic signaling prompts cancer cells to secrete inflammatory factors to surrounding cells, thereby facilitating tumorigenesis. Elevated reactive oxygen species (ROS) in the TME can enhance NADPH production by secreting inflammatory cytokines, stabilizing hypoxia-inducible factor (HIF) and activating the AMPK signaling pathway, thus promoting tumor metastasis and angiogenesis [266]. ROS are also capable of directly inducing oxidative damage to DNA, exemplified by the generation of 8-hydroxy-2′-deoxyguanosine (8-OHdG), and causing gene mutations, such as those in the Ras gene, which may give rise to abnormal cell function and trigger the activation of inflammatory signaling pathways. For example, mutated Ras proteins can lead to the expression of pro-inflammatory cytokines, such as IL-1 and IL-6, and recruit inflammatory cells, to promote angiogenesis [243,267]. This process underscores the pivotal role of ROS not only in causing direct cellular damage but also in fostering an environment conducive to inflammation and tumor progression. Significantly, persistent oxidative stress results in a decline in the activity of antioxidant enzymes (e.g., SOD and GPx) and a reduction in GSH levels, thereby impeding the effective scavenging of ROS. This, in turn, activates pathways, such as NF-κB, and promotes the development of chronic-inflammation-related diseases, such as cancer and cardiovascular diseases [243,251,267]. Notably, oxidative-stress-mediated chronic inflammation not only promotes genetic instability during the early stages of tumorigenesis but also provides a favorable environment for tumor cell survival, facilitating tumor progression by inducing angiogenesis and evading immune surveillance. In addition, there is a feedback loop between ROS and chronic inflammation, which is mainly initiated by immune cells caused by chronic inflammation. These immune cells not only promote chronic inflammation by releasing cytokines but also further aggravate inflammation by producing ROS [11]. In a chronic inflammatory environment, myeloid cells can secrete ROS and inflammatory mediators to aggravate chronic inflammation, suggesting that myeloid cells may play a key role in the inflammatory tumor microenvironment, especially in tumor metastasis [268]. It can be concluded that elevated ROS levels induce oxidative stress and inflammation, which in turn promote the occurrence and development of tumors. Targeting ROS may be an effective strategy for cancer therapy.

Current research suggests that there is a vicious cycle between chronic inflammation and ROS. Chronic inflammation can promote ROS production, and, in turn, ROS can facilitate the formation of chronic inflammation. Inflammation constitutes a pivotal innate defense mechanism against detrimental stimuli, and the inflammatory response triggers massive ROS production to combat pathogens. The defense process is mainly mediated by the activation of immune cells caused by a variety of factors. Activated immune cells produce large amounts of ROS, causing DNA damage, which is a cancer signal [269]. Therefore, ROS, as well as activation of ROS-mediated signaling pathways, may cause oncogenic effects. For example, during the inflammatory cascade, phagocytes, such as neutrophils and macrophages, harness the respiratory burst mechanism to produce ROS as an integral component of their pathogen-elimination strategy [3]. Nevertheless, in chronic inflammation, the overproduction of ROS not only targets foreign invaders but also damages surrounding healthy tissues and exacerbates oxidative stress levels [9]. As an illustration, in IBD, ROS actively participate in the inflammatory response and intensify oxidative stress [8,10]. Significantly, inflammatory responses and oxidative stress exhibit a bidirectional relationship, mutually reinforcing each other and thereby facilitating tumor progression [3,10]. During acute inflammation, immune cells, such as neutrophils and macrophages, deploy the respiratory burst mechanism to generate copious amounts of ROS for pathogen clearance. For example, activated phagocytes like neutrophils and macrophages produce ROS as a key weapon in their arsenal to combat pathogens during the inflammatory response [3,10,139,270]. However, in chronic inflammation, the excessive production of ROS transcends its role in pathogen elimination, causing extensive damage to surrounding normal tissues and escalating oxidative stress. TAMs, which are prominent in the TME, often adopt an M2 phenotype [3,10,138,139,267]. While secreting anti-inflammatory factors, they also release ROS and other oxidants, contributing to the maintenance of oxidative pressure within the TME [271]. In addition, inflammatory mediators produced during the inflammatory response can directly or indirectly promote oxidative stress. For example, pro-inflammatory cytokines, such as TNF-α and IL-1β, can activate NADPH oxidase, a major source of ROS in the body. Moreover, prostaglandins generated during inflammation can disrupt mitochondrial function, diminishing their antioxidant capacity and rendering cells more vulnerable to oxidative damage. Persistent chronic inflammation can lead to repeated cycles of tissue injury and repair during tissue regeneration, leading to the accumulation of genetic mutations and an elevated risk of cancer development. More importantly, the inflammatory microenvironment appears to favor the survival and self-renewal of cancer stem cells, potentially due to the selective pressure exerted by sustained oxidative stress, which promotes the emergence of tumor cell subpopulations with enhanced resistance and adaptability. It is worth noting that during inflammation, white blood cells and mast cells are recruited to the site of injury, triggering a “respiratory burst” that leads to the production and accumulation of large amounts of ROS [272,273]. Zhou et al. showed that all known activators of inflammasomes containing LRR-, NOD-, and NLRP3-based vesicles can induce ROS production. Importantly, studies have demonstrated that the mutagenic effects of ROS generated by inflammatory cells can promote tumor development [274]. Therefore, within the TME, oxidative stress and inflammation synergistically reinforce each other’s effects, creating a vicious cycle that is central to tumor initiation and progression. Both oxidative stress and inflammation are implicated at every stage of tumorigenesis, from genetic mutations and epigenetic alterations to immune escape. A deeper understanding of this intricate relationship not only helps to uncover the molecular mechanisms underlying tumor formation but also provides a theoretical foundation for the development of novel therapeutic strategies. Future research should prioritize the exploration of strategies to effectively disrupt this vicious cycle, with the ultimate goal of preventing and controlling cancer.

## 5. Diagnostic and Therapeutic Implications

### 5.1. Biomarkers

Oxidative-stress- and inflammation-associated tumor biomarkers exhibit multifaceted scientific and clinical relevance in early cancer detection, prognostic assessment, and mechanistic elucidation of tumorigenesis (Table 1). For early screening, noninvasive biomarkers, including 8-OHdG, C-reactive protein (CRP), and gut-microbiota-derived metabolites (e.g., the butyrate-to-deoxycholic acid ratio), serve as molecular indicators of DNA oxidative damage, chronic inflammatory states, and dysbiosis in host–microbiota interactions [275,276,277]. By quantifying these pathophysiological alterations, these markers significantly enhance the sensitivity and specificity of screening protocols for high-risk populations [275]. In prognostic assessment, longitudinal monitoring of the glutathione redox status (GSH/GSSG ratio), lactate levels, and IL-6 concentrations allows for quantitative characterization of spatiotemporally dynamic tumor redox stress and inflammatory microenvironments. This analytical framework enables precise prediction of treatment resistance and recurrence risks by capturing heterogeneous tumor biology [278,279]. Mechanistically, these biomarkers act as molecular sentinels, unveiling core regulatory modules within the oxidative stress–inflammation axis that govern tumor progression—including the ROS–NF-κB positive feedback circuitry and NLRP3 inflammasome activation thresholds [254,274]. Furthermore, these biomarkers illuminate cross-kingdom interaction mechanisms within microbiota metabolic reprogramming–epigenetic modification axes, exemplified by *Fusobacterium nucleatum*-mediated ROS production via the FadA/β-catenin signaling axis [280]. While persistent technical challenges exist in characterizing spatiotemporal heterogeneity and establishing detection standardization, emerging methodologies, including single-cell multi-omics profiling, artificial-intelligence-driven biomarker network modeling, and microbiome-targeted engineering strategies, are propelling the field toward high-resolution, dynamically integrated systems and medicine paradigms. These advancements foster a closed-loop evidence framework that bridges mechanistic discoveries with clinical translation, enabling precision cancer prevention and therapy through iterative refinement of diagnostic and therapeutic strategies.

8-OHdG, a well-characterized biomarker of DNA oxidative damage, arises from ROS-mediated guanine oxidation, directly contributing to genomic instability. This lesion induces mutagenic G → T transversions in key cancer-related genes, including tumor-suppressor loci (e.g., *TP53*) and oncogenes (e.g., *KRAS*) [276]. Mechanistically, 8-OHdG disrupts base excision repair (BER) pathways by inhibiting OGG1 glycosylase activity, thereby escalating the mutational burden in proliferating cells [281]. Accumulated 8-OHdG levels exhibit a strong positive correlation with tumor genomic instability, underscoring its role as a critical driver of mutagenesis. Notably, in chronically inflamed microenvironments, proinflammatory cytokines, such as TNF-α, synergize with ROS to upregulate APOBEC3B deaminase expression, amplifying 8-OHdG-associated mutational signatures by, for example, enriching TCW motif mutations in breast cancer genomes [282]. Furthermore, recent investigations have uncovered that oxidative stress induces extracellular vesicle release via the OGG1–SYT7 signaling axis, thereby facilitating tumor metastasis. As a pivotal mediator of oxidative signaling, 8-OHdG enhances NF-κB pathway activation through epigenetic reprogramming, establishing a self-reinforcing cycle of “oxidative damage–inflammatory activation–metastasis initiation”. In pleural effusion-derived cells from lung cancer patients, 8-OHdG coexists with KRAS and TP53 mutations at frequencies of 64.2% and 69.8%, respectively, findings that provide empirical evidence for its synergistic contribution to tumor progression.

In early cancer detection, 8-OHdG has emerged as a promising noninvasive biomarker with substantial clinical utility. Elevated serum and urinary 8-OHdG concentrations can be detected in prelesional stages, preceding the appearance of imaging-identifiable lesions. For instance, in lung cancer high-risk cohorts, combined measurement of serum 8-OHdG and IL-6 has been shown to significantly enhance early diagnostic sensitivity compared to conventional modalities [276]. Moreover, 8-OHdG levels exhibit a dose-dependent correlation with the malignant potential of pulmonary nodules, supporting their use in risk stratification [283]. In CRC screening, fecal 8-OHdG measurement combined with SDC2/TFPI2 gene methylation detection has been demonstrated to improve the identification rate of advanced adenomas, outperforming traditional fecal occult blood tests in detecting preinvasive lesions [284]. Notably, recent data from China’s National Cancer Center demonstrate a dose-dependent association between occupational exposures (e.g., steelworkers) and both elevated 8-OHdG levels and TP53 mutation frequencies, underscoring their utility in identifying environment-associated tumor risks. In prognostic stratification, dynamic 8-OHdG expression profiles in tumor tissues offer critical insights for predicting treatment resistance and recurrence risk. For CRC patients, low intratumoral 8-OHdG levels correlate with a higher risk of lymph node metastasis and poorer 5-year survival outcomes, whereas high expression is associated with improved sensitivity to chemotherapeutic regimens [285]. In hepatocellular carcinoma (HCC) patients who undergo radical resection, elevated postoperative serum 8-OHdG levels are significantly associated with an increased risk of early tumor recurrence [286]. This clinical correlation is hypothesized to arise from persistent oxidative stress-driven EMT activation within residual micrometastases, facilitating metastatic seeding [287]. Furthermore, 8-OHdG acts as a dynamic biomarker of therapeutic efficacy for guiding antioxidant intervention strategies. By reducing 8-OHdG levels, these therapies restore cellular redox homeostasis, a mechanism corroborated by their demonstrated ability to lower HbA1c levels in diabetic populations [276]. Such findings highlight the translational potential of 8-OHdG-targeted redox modulation as an adjuvant strategy in oncological care.

**Table 1:** Biomarkers related to oxidative stress or inflammation in cancer biology.

Biomarkers	Description of Correlation	Significance in the Occurrence and Development of Tumors	Significance in Clinical Diagnosis and Treatment	References
CRP	Inflammation	High levels of CRP are associated with an increased risk of cancers.	An indicator to evaluate a chronic inflammatory state, it is helpful for cancer risk assessment.	[288,289,290,291,292]
IL-6	Inflammation	Promoting the growth and metastasis of tumor cells and inhibiting the surveillance of tumor cells by the immune system.	It can be used to monitor disease progression and treatment efficacy in patients with cancer.	[293,294,295,296]
COX-2	Inflammation	It induces inflammation by promoting prostaglandin synthesis, which in turn supports tumor cell proliferation, invasion, and angiogenesis.	It can be used as an important reference for tumor diagnosis, prognosis, and treatment target selection.	[297,298,299,300]
MDA	Oxidative stress	Elevated concentrations indicate excessive oxidative stress in the body, which may impair normal cellular structure and function, thus creating the conditions for tumorigenesis.	The detection of MDA can help to understand the individual oxidative stress status and indirectly indicate the risk of cancer.	[301,302,303]
8-OHdG	Oxidative stress	High levels of 8-OHdG mean the DNA has been subjected to more oxidative attack, increasing the probability of genetic mutations.	It is helpful to evaluate the degree of oxidative damage in individuals and has potential value for predicting cancer susceptibility.	[303,304,305]
4-HNE	Oxidative stress	It combines with a variety of biological macromolecules, such as proteins and DNA, leading to cell dysfunction and participating in the occurrence and development of tumors.	The determination of 4-HNE can reflect the lipid peroxidation status in vivo, which is helpful for evaluating the stage of tumor development and its prognosis.	[306,307,308]
SOD	Antioxidant defense system	It can catalyze the disproportionation of superoxide anion free radicals into hydrogen peroxide and oxygen, reduce the damage caused by oxidative stress to cells, and inhibit the formation and development of tumors to a certain extent.	It can be used as an important index to evaluate the antioxidant capacity of the body, and it has guiding significance for tumor prevention and treatment.	[309,310]
GPX	Antioxidant defense system	By reducing peroxides to water or alcohol, cells are protected from oxidative damage and the likelihood of tumorigenesis is reduced.	The level of GPX activity is closely related to the risk and severity of cancer, so it can be used as one of the bases for early warning and intervention measures of cancer.	[310,311,312,313]

Lipid peroxidation-derived metabolites, including malondialdehyde (MDA) and 4-hydroxynonenal (4-HNE), arise from oxidative peroxidation of polyunsaturated fatty acids (PUFAs), a hallmark of oxidative stress. These endogenous electrophilic molecules serve as critical biomarkers of redox dysregulation, exhibiting substantial utility in deciphering tumor progression and prognostic stratification. Mechanistic investigations have established that PUFA peroxidation represents a key mechanistic driver of ferroptosis, an oxidative form of regulated cell death. The resultant MDA and 4-HNE compromise cellular membrane integrity and trigger proinflammatory signaling cascades, thereby amplifying inflammatory responses within the TME [314]. Specifically, MDA covalently binds to guanine residues to form M1G adducts, directly inducing mutagenic G → T transversions in tumor-suppressor genes, such as TP53. Concurrently, MDA inhibits the catalytic activity of key BER enzymes, including OGG1 and APE1, thereby escalating genomic instability. This mechanistic link is supported by clinical evidence demonstrating a positive correlation between serum MDA levels and tumor genomic instability scores in CRC patients [315,316]. In parallel, 4-HNE undergoes covalent modification of mitochondrial cardiolipin (CL), disrupting electron transport chain function and eliciting burst-like ROS production. This bioactive aldehyde further activates proinflammatory signaling axes, including NF-κB and NLRP3 inflammasome pathways, driving the transcriptional upregulation and secretion of proinflammatory cytokines, such as IL-6 and TNF-α. This establishes an “oxidative damage–inflammatory activation” positive feedback circuitry, whereby amplified inflammatory responses drive tumor cell proliferation, invasive potential, and immune evasion capabilities [317,318,319]. Mechanistically, S100P orchestrates acetyl-CoA carboxylase 1 (ACC1)-dependent lipid metabolic reprogramming to promote the accumulation of polyunsaturated fatty acid (PUFA)-derived phospholipids in HCC cells, thereby enhancing the generation of lipid peroxidation byproducts. This metabolic rewiring suppresses ferroptosis and facilitates tumor progression [320]. Additionally, 4-HNE covalently modifies β-catenin, enhancing its nuclear translocation efficiency and inducing EMT in HCC cells [321]. Additionally, 4-HNE is also central to the survival and treatment resistance of cancer stem cells. These cells upregulate ROS detoxification through enhanced GSH biosynthesis, thereby reducing intracellular 4-HNE accumulation and augmenting their tolerance to radiation and chemotherapy [322]. Clinically, elevated serum levels of MDA and 4-HNE correlate significantly with adverse prognoses in multiple solid tumors, including hepatocellular carcinoma, lung cancer, and CRC [323]. Their increased concentrations are directly associated with resistance to targeted agents, such as sorafenib, underscoring their potential as biomarkers for molecular subtyping and therapeutic stratification [320]. Notably, recent investigations have uncovered an inverse correlation between diminished 4-HNE levels and enhanced tumor aggressiveness in non-small cell lung cancer (NSCLC), with such reductions prognosticating poor clinical outcomes. This phenomenon is mechanistically attributed to FOXO4, a tumor suppressor gene whose expression and nuclear activity exhibit a negative correlation with tumor invasiveness and malignant potential. Specifically, reduced intracellular 4-HNE concentrations induce downregulation of FOXO4 expression and impair its transcriptional activity, thereby facilitating uncontrolled tumor cell proliferation and enhancing invasive capacity [324]. Furthermore, lipid-peroxidation-derived metabolites have emerged as critical mediators of synergistic interactions with immunotherapeutic modalities. For example, 4-HNE potentiates the therapeutic efficacy of immune checkpoint inhibitors (ICIs) by augmenting CD8+ T cell effector function and proliferation [279,318]. Notably, pharmacological inhibition of lipid peroxidation through targeting GPX4 or ACSL4 has been shown to overcome tumor resistance to immunotherapy and improve clinical outcomes, thereby presenting a promising strategy for developing combinatorial treatment regimens [318,322]. These cumulative findings collectively establish a robust theoretical framework for integrating lipid peroxidation biomarkers and modulators into precision oncology strategies.

Superoxide dismutase (SOD) and glutathione peroxidase (GPx) represent pivotal antioxidant enzymes with context-dependent bifunctional roles in tumor initiation, progression, and therapeutic response. SOD catalyzes the dismutation of O_2_•^−^ into H_2_O_2_, whereas GPx family members utilize GSH to reduce H_2_O_2_ and organic hydroperoxides, collectively maintaining intracellular redox homeostasis [325,326]. Paradoxically, tumor cells frequently exhibit selective upregulation of SOD and GPx isoforms to evade oxidative-stress-induced apoptosis, thereby facilitating uncontrolled proliferation, enhanced invasive potential, and metastatic dissemination [327,328]. Preclinical evidence demonstrates that genetic or pharmacological downregulation of overexpressed SOD in colorectal and nasopharyngeal cancer models promotes tumor cell apoptosis, suppresses migratory capacity, and inhibits metastatic seeding [329]. GPx4, a pivotal regulator of ferroptosis, attenuates lipid peroxidation upon overexpression, thereby conferring resistance to ferroptosis-inducing agents, such as RSL3 [330]. In cancer stem cells (CSCs), GPx4 maintains lipid peroxidation homeostasis, sustaining stem cell pluripotency and chemotherapeutic resistance; preclinical studies demonstrate that pharmacological or genetic targeting of GPx4 potently suppresses CSC’s self-renewal capacity [331,332]. Clinically, aberrant serum or tissue enzymatic activity of SOD and GPx family members serves as a potential biomarker panel for early cancer detection and prognostic stratification [333]. For example, elevated GPx4 expression in CRC tissues correlates significantly with adverse clinical outcomes and resistance to 5-FU-based chemotherapy [334,335]. Elevated GPx1 levels in pancreatic-cancer-derived exosomes correlate significantly with advanced tumor stages and adverse prognoses [336,337]. Conversely, SOD activity in lung cancer tissues demonstrates a strong association with EGFR mutation status, thereby facilitating molecular subtyping of NSCLC [338]. Pharmacological targeting of SOD and GPx enzymatic activities has emerged as a promising therapeutic strategy to enhance anticancer efficacy. For instance, the SOD mimetic EUK-134, when combined with chemotherapeutic agents, induces synergistic apoptosis in pancreatic cancer cells, while GPx4-specific inhibitors, such as RSL3, trigger ferroptosis and exhibit potentiated anti-tumor effects when paired with sorafenib in hepatocellular carcinoma and other solid malignancies [339,340,341]. Notably, differential expression of SOD and GPx isoforms within the TME is strongly correlated with immunosuppressive signaling landscapes, potentially influencing responses to immune checkpoint inhibitors [342,343]. However, therapeutic modulation of these antioxidant systems necessitates careful mechanistic consideration due to their context-dependent roles. For example, mitochondrial SOD2 suppresses EMT by regulating ROS homeostasis in specific cancer subtypes; conversely, genetic or pharmacological depletion of SOD2 has been shown to exacerbate tumor progression through compensatory redox imbalances in these contexts [344,345]. These findings underscore the intricate tumor type and TME-specific functions of SOD and GPx family members, emphasizing the critical need for precision therapeutic strategies informed by multi-omic profiling and longitudinal biomarker monitoring.

C-reactive protein, a hepatocyte-derived acute-phase reactant, mediates tumor progression by facilitating the establishment of chronically inflamed microenvironments. Elevated systemic CRP levels activate pro-tumorigenic signaling cascades, including NF-κB and MAPK pathways, inducing tumor cells to secrete proinflammatory cytokines, such as IL-6 and TNF-α. These cascades promote angiogenesis, inhibit anti-tumor immune surveillance, and enhance tumor cell invasion and metastatic dissemination [346]. In cancer screening paradigms, CRP levels exhibit a dose-dependent association with malignant transformation risk. A large Japanese prospective cohort study demonstrated that individuals in the highest CRP quartile exhibited a 28% increased risk of overall cancer incidence compared to the lowest quartile, with significant associations observed for colorectal, lung, and breast cancer development [347]. Cancer risk increases in a CRP-concentration-dependent manner, with progressive elevation of systemic CRP levels associated with heightened malignant potential. In NSCLC, preoperative serum CRP levels correlate significantly with tumor invasive characteristics and histological malignancy grade, serving as an adjunctive biomarker to improve early diagnostic accuracy [348]. However, the inherent lack of specificity of CRP limits its utility as a standalone tumor marker, necessitating combinatorial approaches that integrate tumor-specific antigens (e.g., carcinoembryonic antigen, CEA) or advanced imaging techniques to enhance screening precision. In prognostic stratification, CRP functions as an independent prognostic factor; elevated preoperative levels in colorectal cancer patients reflect intensified tumor microenvironmental inflammation, which is strongly associated with increased postoperative recurrence risk and shortened overall survival [349]. In hematological and gastrointestinal malignancies, elevated C-reactive protein/albumin (CRP/ALB) ratios reflect progressive systemic inflammation and malnutrition, which are strongly associated with shortened overall survival [350,351]. Notably, clinical interpretation of CRP must account for tumor histotype and microenvironmental context. The CALLY index, a composite score incorporating CRP, serum albumin, and lymphocyte count, has been validated as an independent prognostic biomarker for digestive system neoplasms. A multicenter clinical investigation involving 7916 patients demonstrated that lower CALLY scores correlate with poorer overall, disease-free, and recurrence-free survival outcomes, with consistent findings across diverse tumor histotypes and demographic populations [351]. However, CRP elevation in pancreatic cancer may complicate differential diagnosis due to overlap with severe acute pancreatitis, necessitating integrated clinical and imaging evaluations. Collectively, these findings establish a theoretical framework for CRP as a broadly applicable tumor-associated inflammatory biomarker while emphasizing the need for prospective validation of its specificity and sensitivity in large-scale, multi-center trials.

COX-2, a key enzyme in the arachidonic acid pathway, catalyzes the conversion of arachidonic acid into bioactive eicosanoids, including prostaglandins (PGs) and thromboxanes, which are central to regulating inflammatory responses and tissue homeostasis [352]. While exhibiting low basal expression in most normal tissues, COX-2 is aberrantly overexpressed in a wide spectrum of malignancies, including lung, gastrointestinal, breast, and hepatocellular carcinomas. This dysregulated expression drives carcinogenesis through multiple mechanisms: excessive production of pro-inflammatory prostaglandin E2 (PGE2) promotes tumor cell proliferation, inhibits apoptosis, and facilitates angiogenesis; concurrently, COX-2-mediated remodeling of the TME induces immunosuppressive signaling, reduces anti-tumor immune surveillance, and enhances metastatic potential [353]. Clinically, COX-2 overexpression is associated with increased recurrence risk, shortened overall survival, and resistance to chemotherapeutic and radiotherapeutic interventions, solidifying its role as a pivotal oncogenic driver across diverse cancer types. COX-2-catalyzed synthesis of PGE2 triggers a cascade of pro-tumorigenic events by engaging EP2/4 prostanoid receptors. This ligand-receptor interaction activates NF-κB and STAT3 signaling axes, concurrently inhibiting p53-mediated apoptotic pathways and upregulating secretion of proinflammatory cytokines such as IL-6 and TNF-α. Collectively, these processes establish a chronically inflamed TME that promotes uncontrolled tumor cell proliferation, invasive migration, and EMT [354]. Furthermore, COX-2-mediated immune evasion occurs through dual mechanisms: suppression of macrophage phagocytic activity dampens anti-tumor immune surveillance, while VEGF upregulation drives angiogenesis in CRC, facilitating metastatic niche formation [355]. In cancer risk assessment, elevated COX-2 expression has been causally linked to increased susceptibility to colorectal and lung carcinogenesis. A 2022 prospective cohort study by Japan’s National Cancer Center demonstrated that COX-2, as a mechanistic inflammatory biomarker, enhances risk stratification for tumor initiation when integrated with traditional markers like CRP, providing incremental value beyond standalone CRP assessment [347]. In a landmark study, Keisuke Kosumi, et al. employed Cox proportional hazards regression to analyze data from 1,708 stage I–IV CRC patients, revealing a statistically significant interaction between COX-2 expression and BRAF mutation status. Among BRAF-mutant tumors, high COX-2 expression was independently associated with significantly poorer overall survival compared to COX-2-negative counterparts, whereas this prognostic association was absent in BRAF-wild-type cases. These findings validate COX-2 as a robust prognostic biomarker specific to BRAF-mutant CRC, underscoring its utility in molecular subtyping for personalized risk stratification [356]. Clinically, selective COX-2 inhibitors such as celecoxib have demonstrated chemopreventive and therapeutic benefits: long-term use is associated with reduced incidence of colorectal, lung, esophageal, and hepatic malignancies, while adjuvant administration enhances response to cytotoxic chemotherapy by sensitizing tumor cells to apoptosis. Mechanistic studies attribute these effects to COX-2-mediated suppression of pro-inflammatory prostaglandin signaling and modulation of drug resistance pathways [357,358]. Combination strategies integrating COX-2 inhibitors with cytotoxic chemotherapeutics (e.g., alkylating agents, antimetabolites, anti-tumor antibiotics), monoclonal antibodies, and other antineoplastic agents are being actively investigated to optimize therapeutic efficacy while minimizing treatment-related toxicities [358]. Indeed, the FDA has approved celecoxib for the management of familial adenomatous polyposis (FAP), and other COX-2 inhibitors are also being explored for cancer treatment and prevention. Meanwhile, mechanistic investigations further reveal synergistic interactions with conventional cytotoxins. For example, aspirin enhances cisplatin sensitivity in colorectal cancer cells by disrupting NF-κB binding to the *COX-2* promoter, thereby suppressing COX-2-dependent pro-survival signaling [359]. These findings underscore the translational promise of targeting the COX-2-inflammatory axis as part of multi-modal cancer treatment strategies, emphasizing the need for biomarker-driven patient selection to maximize clinical benefit.

Furthermore, a multitude of additional tumor biomarkers associated with oxidative stress and inflammatory pathways have been implicated in critical roles across tumor pathogenesis, progression, and prognostic evaluation. The persistent activation of NF-κB represents a core component of the oxidative stress–inflammation axis, serving to regulate the expression of pro-inflammatory mediators such as IL-6 and COX-2. In HCC and gastric cancer, dysregulated NF-κB signaling has been consistently linked to enhanced tumor cell proliferation and chemoresistance, whereas pharmacological inhibition of NF-κB has been shown to augment the therapeutic efficacy of 5-FU [360]. Nrf2, a pivotal transcription factor governing antioxidant responses, exerts biphasic roles in carcinogenesis: its activation during the initiation phase suppresses tumorigenesis by upregulating antioxidant genes, whereas sustained activation at advanced stages promotes chemoresistance through enhanced GSH biosynthesis and upregulation of multidrug efflux transporters [351]. Clinical evidence demonstrates that elevated Nrf2 expression is associated with cisplatin resistance in NSCLC and sorafenib resistance in HCC. Consequently, combinatorial strategies targeting both antioxidant depletion and Nrf2 inhibition may potentiate chemotherapeutic sensitivity by disrupting adaptive resistance mechanisms [361,362]. Additionally, elevated Nrf2 expression is associated with poor prognosis in non-small cell lung cancer (NSCLC), and the activation status of Nrf2 in KEAP1-mutant lung cancer patients serves as a predictor of tumor sensitivity to antioxidant therapies [361,362]. *Fusobacterium nucleatum* drives tumorigenesis through the FadA adhesin, which activates the β-catenin/NF-κB signaling axis to induce the production of ROS [363,364]. High *F. nucleatum* abundance in CRC tissues has been shown to correlate with resistance to 5-FU [365]. Detection of this bacterium in fecal samples is emerging as a potential biomarker for early-stage CRC screening. Moreover, metabolites derived from the gut microbiota, such as butyrate and secondary bile acids, are implicated in critical regulatory roles during tumor progression. Butyrate exerts anti-tumor effects by inhibiting histone deacetylases (HDACs), thereby enhancing the expression of the transcription factor ID2 in CD8+ T cells and potentiating anti-tumor immune responses [366,367,368]. In HCC, butyrate suppresses tumor cell proliferation and metastasis while augmenting the therapeutic efficacy of sorafenib [367]. However, its biological actions are contingent upon microbial origin and concentration. For instance, *Bifidobacterium*-derived butyrate inhibits non-alcoholic fatty liver disease-associated HCC development, whereas butyrate generated under a high-fructose diet activates pro-inflammatory signaling through the SCFA receptor GPR43, thereby promoting HCC progression [366,367,368]. Secondary bile acids, including deoxycholic acid (DCA), promote CRC cell proliferation and EMT by activating the farnesoid X receptor (FXR)-NF-κB signaling axis. Conversely, lithocholic acid (LCA), a secondary metabolite derived from cholic acid, inhibits the activation of the NLRP3 inflammasome, thereby reducing the secretion of pro-inflammatory cytokines IL-1β and TNF-α. This dual action alleviates intestinal inflammation and diminishes the risk of CRC development [366,367,368].

### 5.2. Therapeutic Strategies Targeting Oxidative Stress–Inflammation Axis

#### 5.2.1. Oxidative-Stress-Based Cancer Intervention Strategies

In cancer therapeutics, redox homeostasis modulation strategies encompass two primary approaches: ROS-depleting therapies that employ antioxidant agents, and ROS-promoting strategies that elevate intracellular ROS levels through direct or indirect inhibition of cellular antioxidant systems [16,369]. In normal somatic cells, endogenous antioxidant mechanisms serve to mitigate toxic free radical-induced damage and safeguard against malignant transformation. Conversely, within established neoplastic tissues, the multifaceted role of oxidative stress in tumor initiation, progression, and microenvironmental adaptation highlights the limitations of oversimplified ROS-modulating therapeutic paradigms. In the context of cancer biology, while ROS are implicated in driving neoplastic progression, initial hypotheses proposing that antioxidant therapies could suppress tumors have not been unequivocally validated. Emerging evidence instead highlights that pharmacological ROS suppression may inadvertently facilitate cancer progression and enhance treatment resistance mechanisms [7,33]. Mechanistically, if ROS-mediated carcinogenesis proceeds via induction of mutagenesis and by acting as second messengers in cell proliferation signaling pathways, antioxidant intervention to mitigate oxidative stress could theoretically impede tumor initiation, a premise underpinning the development of certain antioxidant-based cancer therapies. However, clinical and preclinical data indicate that antioxidant drugs, by scavenging ROS, may attenuate the cytotoxic efficacy of established chemotherapeutics and radiotherapy [370,371]. Moreover, dietary strategies incorporating natural antioxidants have been explored for cancer prophylactic applications (Table 2). Nutrient components with antioxidant activities, including vitamins A/D, genistein, sulforaphane, curcumin, tannins, and flavonoids, have demonstrated potential to inhibit tumor initiation and progression in breast, liver, and pancreatic cancers, among others [29,372,373]. A landmark large-scale randomized double-blind trial examining the chemopreventive effects of selenium, vitamin E, and β-carotene supplementation reported that this combination significantly reduced all-cause mortality in esophageal and gastric cancer patients, with protective effects persisting for over a decade following intervention discontinuation [374,375]. Concurrently, epidemiological evidence has established an inverse association between long-term vitamin E intake and HCC risk, with dose-dependent reductions in incidence observed across prospective cohort studies [376]. Paradoxically, longitudinal investigations in smoking populations revealed that sustained supplementation with β-carotene alongside vitamins A/E was associated with elevated lung cancer incidence, overriding any putative chemopreventive effects in this high-risk subgroup [377,378]. While initial reports suggested selenium may mitigate prostate cancer progression, subsequent mechanistic studies demonstrated that this protective effect is exclusively confined to aggressive metastatic phenotypes and exhibits strong dependence on genetic polymorphisms in selenoprotein-coding genes [379]. A large-scale epidemiological study involving 35,533 males with a long-term follow-up period demonstrated that supplementation with selenium or vitamin E in healthy populations failed to show a significant reduction in prostate cancer risk. Conversely, prolonged intake of vitamin E was associated with an increased risk of prostate cancer among healthy males [380]. These conflicting findings underscore the intricate dynamics of redox balance in determining cell fate within normal versus neoplastic cellular microenvironments. Future research and translational strategies must incorporate molecular subtyping of tumors, individual metabolic profiles, and interindividual variability to develop precision-oriented antioxidant strategies that optimize outcomes in cancer prevention and therapeutic interventions.

Elevated ROS levels disrupt cellular homeostasis, whereas oxidative stress can exert tumor-suppressive effects, driving cancer cells to evolve robust antioxidant defense mechanisms for adaptive survival. Despite upregulating intracellular antioxidant systems, cancer cells maintain higher basal ROS levels than normal cells. This metabolic disparity establishes a therapeutic window, as malignant cells may exhibit heightened sensitivity to agents that further induce ROS accumulation [29]. Indeed, numerous clinically approved chemotherapeutic compounds exert their cytotoxic effects by elevating ROS levels (Table 2), although such treatments inherently carry risks of promoting off-target tumorigenesis. Taxanes (paclitaxel, docetaxel), vinca alkaloids (vincristine, vinblastine), and antimetabolites (fluorouracil, fludarabine) act through dual mechanisms: inducing cytochrome c release from mitochondria to trigger apoptotic cell death and disrupting mitochondrial electron transport chains to generate free radicals [388,390,394]. Anthracyclines, including doxorubicin, epirubicin, and daunorubicin, exert anti-tumor effects by inducing excessive ROS and RNS, which trigger DNA damage, mitochondrial dysfunction, and impaired protein synthesis [391]. Arsenic trioxide (As_2_O_3_), 2-methoxyestradiol, and N-(4-hydroxyphenyl)-retinamide (4-HPR) mediate apoptosis across multiple cancer types, including leukemia, multiple myeloma, and breast cancer, via ROS overproduction [383,384,385]. Additionally, platinum-based anticancer agents (cisplatin, carboplatin, oxaliplatin) induce oxidative stress through their high-affinity binding of platinum (Pt) moieties to sulfur and selenium atoms in proteins/peptides, thereby disrupting key cellular antioxidant systems such as GSH and thioredoxin reductase (TrxR) [395]. Cisplatin resistance is primarily mediated by its covalent interaction with GSH, as GSH readily forms conjugates with the drug’s platinum center, thereby facilitating drug detoxification [396]. Paradoxically, this detoxification process depletes intracellular GSH stores, disrupting redox homeostasis and inducing ROS overproduction. Notably, elevated GSH levels in neoplastic cells correlate with resistance not only to cisplatin but also to anthracyclines and alkylating agents. Additionally, tumors adapting to chronic oxidative stress through upregulation of SOD2, peroxidase 1, and B-cell lymphoma-2 (BCL-2) protein exhibit enhanced chemoresistance to agents like 5-FU [393]. Targeting tumor redox homeostasis, via inhibition of GSH/SOD2 biosynthesis or induction of their metabolic depletion, represents a promising therapeutic strategy to potentiate anticancer drug efficacy [16]. NOV-002, a glutathione disulfide (GSSG) mimetic compound, modulates the intracellular GSSG/GSH redox balance to induce oxidative stress, thereby inhibiting tumor cell invasion, proliferation, and survival [386]. In HER2-negative breast cancer patients, adjuvant therapy combining NOV-002 with doxorubicin, cyclophosphamide, and docetaxel chemotherapy improved clinical outcomes with reduced treatment-related toxicity compared to chemotherapy alone [397]. Poly(ADP-ribose) polymerase (PARP), an enzyme frequently overexpressed in diverse malignancies, plays a pivotal role in preserving genomic integrity and mediating the cellular response to oxidative stress. PARP inhibitors, when combined with platinum-based chemotherapeutics, disrupt cancer cells’ adaptive responses to oxidative stress by impairing DNA repair capacity [398]. Phase II clinical trials demonstrated that triple-negative breast cancer (TNBC) patients derived significant benefit from the combination of the PARP inhibitor veliparib and carboplatin, exhibiting improved progression-free survival [399,400]. Similarly, preclinical studies in Brca1/2-deficient breast cancer mouse models showed that olaparib, a selective PARP inhibitor, enhanced the anti-tumor efficacy of platinum-based therapies, leading to profound tumor growth suppression [401,402]. Furthermore, PARP inhibition has been shown to sensitize NSCLC cells to cisplatin, representing a promising strategy for overcoming chemoresistance in this tumor type [403].

Cancer therapies targeting oxidative stress exert effects beyond direct cytotoxicity toward cancer cells, as they can profoundly influence tumor progression and metastatic potential by modulating stromal remodeling. The nature and subcellular localization of ROS exhibit intricate associations with tumor survival and metastatic dissemination across diverse cancer subtypes, highlighting that single-target interventions tested in simplified experimental settings, while valuable for mechanistic dissection, may fail to recapitulate therapeutic responses in the context of the complex TME. Cancer-associated fibroblasts (CAFs), localized at tumor-stromal interfaces or infiltrating neoplastic tissues, are critical drivers of cancer initiation, progression, and metastasis [404,405]. High CAF density within tumors correlates with adverse clinical outcomes, enhanced infiltration of tumor-associated macrophages, and promotion of EMT [404,405]. Long-term radiotherapy or chemotherapy promotes the activation of CAFs within the TME by enhancing mitochondrial ROS production [406,407]. Chemotherapy-induced ROS or antioxidant depletion may further promote the immunosuppressive polarization of tumor-associated macrophages, thereby fostering an immunosuppressive niche [406,407]. These findings underscore the necessity of integrating both the direct cytotoxic effects of ROS on cancer cells and the indirect pro-tumorigenic impacts mediated by ROS in stromal components during therapeutic strategy design. Notably, oxidative stress-driven mechanisms exhibit context-dependent effects across tumor types: preclinical evidence demonstrates that ROS suppress metastasis in lung cancer models while facilitating metastatic progression in pancreatic cancer models [408,409]. These contextual discrepancies likely arise from inherent variations in ROS biology, encompassing ROS species, subcellular localization, cellular redox responsiveness, and the microenvironmental presence of ferroptosis-regulatory pathways [26,410]. Mechanistic studies demonstrate that mitochondrial ROS act as well-characterized inducers of regulated cell death, whereas nitrogen oxide derived ROS species are increasingly implicated in promoting cell proliferation and migratory phenotypes [7,321]. These findings highlight the need for caution in deploying ROS-modulating therapies, as inappropriate use may not only abrogate therapeutic efficacy but also potentiate malignant progression. Notably, cancer cells exhibit distinct deviations from normal cellular ROS homeostasis, including differential exposure thresholds and stress response dynamics. Continued mechanistic dissection of these context-dependent complexities will be pivotal for developing precision-targeted interventions that leverage ROS signaling or its regulatory networks to selectively address specific cancer vulnerabilities.

#### 5.2.2. Inflammation-Based Cancer Intervention Strategies

Inflammatory cells and their secreted mediators, including cytokines and chemokines, constitute a critical component of the TME, orchestrating processes central to tumor survival, immune cell infiltration, and evasion of immunosurveillance [271]. Modulating inflammatory and immune signaling has thus emerged as a pivotal dimension of cancer prevention and therapeutic strategies (Table 3). Substantial evidence supports the chemopreventive effects of non-steroidal anti-inflammatory drugs (NSAIDs) across multiple cancer types, including colorectal, breast, and esophageal malignancies. Both epidemiological observations and randomized controlled trials demonstrate that regular aspirin use confers significant cancer risk reduction, most pronounced for CRC, while improving clinical outcomes in diagnosed patients by lowering 5-year mortality and disease recurrence rates [17,358]. Combination therapy with aspirin and esomeprazole has been shown to significantly improve clinical outcomes in patients with Barrett’s esophagus while mitigating the risk of esophageal cancer development [411]. NSAIDs exert their chemopreventive effects primarily by inhibiting COX-2 activity, thereby reducing prostaglandin biosynthesis. Prostaglandins mediate pro-inflammatory signaling cascades and promote tumor cell survival, angiogenesis, and immune evasion, which are key processes driving malignant progression. The FDA has approved celecoxib, a selective COX-2 inhibitor, for the treatment of familial adenomatous polyposis [340]. Clinical trials further demonstrate that celecoxib combined with cytotoxic chemotherapy (5-fluorouracil, leucovorin, irinotecan) constitutes a safe and efficacious synergistic regimen for metastatic CRC. Compared with monotherapy, this combinatorial approach improves progression-free survival and one-year overall survival rates, highlighting the potential of inflammatory pathway modulation in enhancing anticancer efficacy [412]. Celecoxib in combination with paclitaxel enhances dendritic cell maturation, thereby triggering T-cell-dependent anti-tumor immune responses and augmenting immune-mediated rejection of TNBC. This synergistic interaction leads to suppressed tumor recurrence and reduced lung metastasis in preclinical models [413]. Clinically, COX-2-high tumor patients derive greater benefit from celecoxib-based combination therapies, as evidenced by significantly improved prognostic outcomes compared to COX-2-low counterparts [414]. A phase III clinical trial in resected stage III CRC patients found no overall statistically significant improvement in disease-free survival (DFS) with celecoxib monotherapy [415]. However, stratified analysis by PIK3CA mutation status revealed a notable subgroup effect: patients harboring PIK3CA gain-of-function mutations experienced prolonged DFS when treated with celecoxib, whereas those with wild-type PIK3CA derived no such benefit [415]. An adjuvant therapy trial evaluating celecoxib in ERBB2-negative breast cancer patients found no significant improvement in DFS following two years of treatment as an add-on to standard therapy [416]. Furthermore, preclinical and clinical evidence suggests that adjuvant celecoxib added to chemotherapy regimens may exert detrimental effects on clinical outcomes in breast cancer subsets, particularly in tumors with low prostaglandin-endoper oxide synthase 2 (PTGS2) expression [417]. These divergent findings underscore the intricate interplay between anti-inflammatory pathways and tumor-specific biological contexts, highlighting the challenges posed by mechanistic complexity and interpatient heterogeneity in translating COX-2-targeted strategies into clinical benefit.

Tumor-associated macrophages represent a dominant immunocyte population within the TME, playing a pivotal role in promoting tumor progression and mediating multidrug resistance [418]. Therapeutic strategies targeting TAMs primarily involve two complementary approaches: direct induction of macrophage apoptosis or inhibition of their recruitment to the tumor niche, and reprogramming their functional polarization to elicit a cytotoxic anti-tumor phenotype (Table 3) [419]. The CCL2/CCR2 chemokine axis is central to orchestrating the recruitment, survival, and expansion of inflammatory monocytes, TAMs, and MDSCs [420]. While preclinical and early clinical data show that CCR2 inhibitors as single agents exhibit modest therapeutic efficacy, their combinatorial use with chemotherapy or immune checkpoint inhibitors has emerged as a promising strategy, leveraging synergistic interactions to enhance anti-tumor immunity and overcome resistance. Monoclonal antibodies targeting CCL2 have been shown to effectively reduce macrophage accumulation in preclinical tumor models and enhance anti-tumor efficacy when combined with conventional chemotherapy [421,422]. A Phase I clinical trial demonstrated that PF-04136309, a small-molecule CCR2 inhibitor, was well-tolerated when integrated into FOLFIRINOX chemotherapy regimens for patients with borderline resectable or locally advanced pancreatic cancer, establishing the safety and feasibility for combinatorial approaches [423]. In pancreatic ductal adenocarcinoma (PDAC) mouse models, the dual CCR2/5 inhibitor BMS-687681, when combined with αPD-1 immunotherapy and radiation, reprogrammed the TME by augmenting intratumoral infiltration of effector T cells and memory T cells while reducing accumulation of Tregs, M2-polarized TAMs, and MDSCs. This multimodal strategy exhibited significantly enhanced anti-tumor activity compared to single-agent or dual-combination regimens in preclinical testing [138]. Bisphosphonates, which exert cytotoxic effects on macrophages while modulating bone remodeling, are clinically utilized for treating postmenopausal osteoporosis and managing bone metastases in cancer patients [424]. In breast and prostate cancer populations, bisphosphonate monotherapy or combination with hormone therapy has been shown to significantly reduce disease recurrence rates and improve overall survival [424]. Trabectedin, a marine-derived alkaloid with selective cytotoxicity toward monocyte-derived macrophages, exhibits multifunctional anti-tumor activities: it inhibits pro-inflammatory mediator secretion, reduces TAMs density within the TME, and suppresses angiogenesis. This agent has been approved by regulatory authorities for the treatment of advanced soft tissue sarcoma and ovarian cancer [425]. Additionally, therapeutic strategies aimed at reprogramming TAMs from an immunosuppressive M2 phenotype to a cytotoxic M1 phenotype represent a promising frontier in cancer immunotherapy, leveraging macrophage plasticity to enhance anti-tumor immune responses. Preclinical studies have shown that the agonistic anti-CD40 antibody CP-870,893 reprograms TAMs into M1-like effector cells with enhanced antigen-presenting capacity, thereby restoring tumor immune surveillance and promoting tumor regression in murine models [426]. Phase I clinical trials demonstrated that combination therapy with anti-CD40 antibody and gemcitabine provided clinical benefit to select patients with advanced pancreatic cancer, highlighting the feasibility of TAMs-targeted immune modulation [427]. The immune checkpoint axis formed by SIRPα, expressed on phagocytes including TAMs, and CD47 on tumor cells inhibits intracellular signaling cascades, thereby negatively regulating phagocytic activity and enabling tumor cells to evade immune elimination. Monoclonal antibodies targeting CD47 have been shown to block this immunosuppressive interaction, successfully inducing antibody-dependent cellular phagocytosis of tumor cells in diverse preclinical tumor models [428,429]. However, these findings underscore the therapeutic potential of CD47 blockade, clinical translation remains contingent on robust validation through ongoing and future clinical trials.

Proinflammatory cytokines, such as tumor necrosis factor (TNF), IL-6, and IL-1β, typically drive tumor progression by enhancing tumor cell proliferation, survival, and invasive potential, as well as promoting angiogenesis [430]. Current therapeutic strategies targeting pro-tumorigenic cytokine signaling largely depend on antibody-based interventions (Table 3). However, monotherapeutic approaches using anti-cytokine antibodies have generally demonstrated suboptimal efficacy, characterized by limited clinical responses and treatment-associated toxicities [138]. Even in multiple myeloma, where IL-6 is recognized as a critical growth factor driving tumor cell proliferation and survival, the clinical efficacy of anti-IL-6 mAbs has been disappointing—despite anecdotal reports of transient benefits in select patient subgroups [208]. This suboptimal response necessitates the integration of these biologics with conventional chemotherapies or targeted agents. For instance, clinical investigations have demonstrated that anti-IL-1β mAbs enhance the therapeutic efficacy of PD-1 checkpoint inhibitors in breast cancer patients [431]. Preclinical studies using renal cell carcinoma mouse models further revealed that combinations of IL-1β inhibitors with anti-PD-1 antibodies or tyrosine kinase inhibitors yielded significantly enhanced anti-tumor responses compared to monotherapeutic approaches [432]. Siltuximab, a chimeric monoclonal antibody targeting IL-6, exhibited acceptable safety profiles and improved clinical outcomes when combined with mitoxantrone/prednisone (M/P) in patients with castration-resistant prostate cancer (CRPC) with prior chemotherapy exposure [433]. Infliximab, a monoclonal antibody against TNF-α, in combination with oxaliplatin has been shown to partially reverse oxaliplatin resistance in CRC, potentially through antibody-dependent cellular cytotoxicity and complement-dependent cytotoxicity mechanisms [434]. Additionally, interferon-α (IFN-α), an FDA-approved adjuvant therapy for melanoma, has been shown to improve DFS and OS in patients who underwent surgical resection [435]. However, select clinical trials have reported suboptimal outcomes, potentially compromising patient health and quality of life [436]. Indeed, cytokine-based anti-tumor therapies remain in the translational development phase, necessitating continued preclinical and clinical investigations to enhance therapeutic windows and mitigate treatment-related toxicities.

**Table 3:** Anticancer treatments according to their role in regulating inflammation.

Name	Mechanism of Action; Effects on Inflammation	Effect on Tumors	References
NSAIDs	Inhibit COX-2 activity, reduce prostaglandin biosynthesis	Reduce the incidence of cancers; inhibit tumor cell survival, angiogenesis, and immune evasion; improve clinical outcomes	[17,358]
CCR2 inhibitors (PF-04136309, BMS-687681)	Cause remodeling of immune cells within the tumor	Enhance anti-tumor immunity and overcome resistance	[138]
Bisphosphonates	Cytotoxic effects on macrophages	Reduce breast and prostate cancer patients’ recurrence rates and improve overall survival	[424]
Trabectedin	Cytotoxicity toward monocyte-derived macrophages; inhibits pro-inflammatory mediator secretion	Inhibit advanced soft tissue sarcoma and ovarian cancer	[425]
CP-870,893	Reprograms TAMs into M1-like effector cells	Facilitate the depletion of tumor stroma; inhibit tumor growth	[426]
Anti-IL-1β mAbs	A monoclonal antibody against IL-1β	Combined treatment enhances the efficacy of chemotherapy drugs	[431,432]
Siltuximab	A chimeric monoclonal antibody targeting IL-6	Combined therapy enhances the efficacy of chemotherapy drugs and improves their safety	[433]
Infliximab	A monoclonal antibody against TNF-α	Reverse oxaliplatin resistance in CRC	[434]
Interferon-α	Regulate the differentiation and infiltration of immune cells	Improve DFS and OS in patients	[435]

## 6. Conclusions

Oxidative stress and inflammation serve as dual drivers of tumorigenesis, functioning synergistically through intricate regulatory networks to promote tumor initiation, clonal expansion, invasive phenotype acquisition, and therapeutic resistance. Oxidative stress disrupts cellular redox homeostasis via the excessive accumulation of ROS, thereby inducing DNA adduct formation, genetic mutations, and epigenetic reprogramming. These molecular perturbations activate pro-oncogenic signaling cascades while concurrently stimulating the secretion of proinflammatory cytokines, thereby establishing a self-reinforcing “oxidative stress–inflammation” positive feedback loop. Proinflammatory cytokines further promote tumor cell survival, angiogenesis, and formation of immunosuppressive TME, thereby driving progressive tumor malignancy. During cancer therapy, oxidative stress and inflammation exhibit bifunctional roles: on one hand, many chemotherapeutic and radiotherapeutic modalities depend on elevating ROS levels to induce tumor cell death. Conversely, cancer cells frequently acquire resistance by upregulating intrinsic antioxidant defense mechanisms, leading to progressive therapy tolerance. In the context of immunotherapy, oxidative stress and the inflammatory TME exert profound effects on effector immune cell functionality and tumor immune evasion capacity. Concurrently, emerging therapeutic strategies aim to modulate the oxidative stress–inflammation axis, encompassing antioxidant supplementation, immunomodulatory pharmacotherapies, targeted inhibition of proinflammatory signaling pathways, and combined intervention. These approaches offer diverse translational opportunities, including cancer risk reduction, enhancement of chemotherapeutic sensitivity, and improvement of clinical prognoses. Preliminary findings from select clinical trials have demonstrated encouraging efficacy, presenting novel therapeutic prospects for patients. Nevertheless, existing therapeutic challenges primarily arise from the intricate nature of oxidative stress- and inflammation-driven tumorigenic mechanisms, coupled with the pleiotropic effects of therapeutic targets. Given the suboptimal outcomes of numerous current approaches, there is an imperative need for in-depth investigations to elucidate these complex pathways and optimize targeted therapeutic strategies.

In conclusion, the development of optimized therapeutic strategies targeting oxidative stress and inflammation in oncology poses significant future challenges. First, there is a critical need for precise elucidation of the spatiotemporal-specific crosstalk mechanisms between oxidative stress and inflammation to inform precision-targeted interventions. Second, ongoing efforts must focus on optimizing existing treatment regimens to achieve balanced modulation of these interconnected pathways, while mitigating potential adverse effects, such as impairment of host immune defenses and diminished resistance to infections, that arise from dysregulated redox-inflammatory homeostasis. Third, intensifying biomarker discovery efforts is essential to establish a scientific basis for personalized therapeutic approaches. Notwithstanding these challenges, breakthroughs in multi-omics methodologies now afford comprehensive mechanistic insights into the dynamic crosstalk between oxidative stress and inflammation within the TME [437]. Concurrently, innovative nanomaterial-based drug delivery platforms show substantial promise in surmounting target specificity limitations, enabling site-specific high-concentration drug release, and enhancing treatment efficacy [438]. Ultimately, the elucidation of the synergistic mechanisms underpinning oxidative stress and inflammation presents novel therapeutic targets for oncological interventions. Nevertheless, realizing precision and personalized cancer prevention and treatment strategies necessitates a fundamental paradigm shift from traditional “single-agent inhibition” towards “systemic regulation” approaches. This transformative shift is predicated on three key pillars: in-depth mechanistic understanding, precision molecular subtyping, and technological advancements. Such an integrative approach will be pivotal for maximizing therapeutic efficacy and improving patient outcomes in cancer management.

## Figures and Tables

**Table 2:** Anticancer treatments according to their role in regulating ROS levels.

Name	Mechanism of Action; Effects on Oxidative Stress	Effect on Tumors	References
β-carotene	Antioxidant effect	Reduce all-cause mortality in esophageal and gastric cancer patients	[374,375]
Vitamins A/D/E	Antioxidant effect	Reduce incidence of cancers and improve patient outcomes	[376,381,382]
Selenium	Antioxidant effect	Mitigate prostate cancer progression	[379]
Nutrient components (genistein, sulforaphane, curcumin, tannins, flavonoids, etc.)	Antioxidant effect	Inhibit the survival of breast, liver, and pancreatic cancers	[29,372,373]
As_2_O_3_	Reacts with cysteine residues, inhibits mitochondrial respiratory function, induces ROS overproduction	Induce apoptosis across multiple cancer types, including leukemia, multiple myeloma, and breast cancer	[383,384,385]
NOV-002	Glutathione disulphide mimetic; alters intracellular GSSG/GSH ratio	Inhibit tumor cell invasion, proliferation, and survival	[386]
Taxanes (paclitaxel, docetaxel)	Downregulation of antioxidant glutathione peroxidase and glutathione	Induce tumor cell apoptosis; inhibit angiogenesis, cell proliferation, and drug resistance	[387,388]
Vinca alkaloids (vincristine, vinblastine)	Induce an imbalance in the cellular oxidation/antioxidant state	Inhibit angiogenesis and tumor growth	[389]
Antimetabolites (fluorouracil, fludarabine)	Induce the generation of free radicals	Induce cancer cell apoptosis and inhibit the progression of CRC	[383,390,391]
Anthracyclines (doxorubicin, epirubicin, daunorubicin)	DNA Integration and Topoisomerase II inhibition; induce excessive ROS and RNS	Have a wide range of anti-tumor effects	[384,392]
Platinum-based anticancer agents (cisplatin, carboplatin, oxaliplatin)	Disrupt cellular antioxidant systems	Induce DNA damage, cycle arrest, and apoptosis in tumor cells	[393]

## Abbreviations

The following abbreviations are used in this manuscript. TME, Tumor microenvironment; ROS, Reactive oxygen species; RNS, Reactive nitrogen species; MAPK, Mitogen-activated protein kinase; HIFs, Hypoxia-inducible factors; TNF-α, Tumor necrosis factor α; IL-6, Interleukin-6; Tregs, Regulatory T cells; MDSC, Myeloid-derived suppressor cells; EMT, Epithelial–mesenchymal transition; IL-1β, Interleukin-1β; ALRS, AIM2-like receptors; NADPH, Nicotinamide adenine dinucleotide phosphate; CRC, Colorectal cancer; COX-2, Cyclooxygenase-2; NAC, N-acetylcysteine; SODs, Superoxide dismutases; PDGF, Platelet-derived growth factor; EGF, Epidermal growth factor; TGFβ, Transforming growth factor β; TCA, Tricarboxylic acid; DNMT, DNA methyltransferase; TSG, Tumor suppressor gene; 5-LO, 5-lipoxygenase; NOX4, NADPH oxidase 4; PTEN, Phosphatase and tensin homolog; CCS, Copper chaperone for superoxide dismutase; ECM, Extracellular matrix; ccRCC, Clear cell renal cell carcinoma; MMP2, Matrix metalloproteinase-2; AURKA, Aberrant expression of serine-threonine kinase A; OSCC, Oral squamous cell carcinoma; E-cadherin, Epithelial cadherin; GSH, Glutathione; TrxR, Thioredoxin reductase; GST, Glutathione S-transferase; AREs, Antioxidant response elements; Keap1, Kelch-like ECH-associated protein 1; GST, Glutathione S-transferase; HO-1, Heme oxygenase-1; AhR, Aryl hydrocarbon receptor; C300, Cysteine at position 300; PTG, Glycogen-targeting protein; PPP, Pentose phosphate pathway; ABC, ATP-binding cassette; MDR, Multidrug resistance; ER, Endoplasmic reticulum; IP3R, Inositol 1,4,5-trisphosphate receptors; GBM, Glioblastoma; HGSOC, High-grade serous ovarian cancer; MIF, Macrophage migration inhibitory factor; TAMs, Tumor-associated macrophages; TANs, Tumor-associated neutrophils; NETs, Neutrophil extracellular traps; HNSCC, Head and neck squamous cell carcinoma; ADCC, Antibody-dependent cell-mediated cytotoxicity; IL-1, Interleukin-1; TNFR2, TNF receptor 2; IFN-I, Type I interferons; IBD, Inflammatory bowel disease; DAMPs, Damage-associated molecular patterns; 8-OHdG, 8-hydroxy-2′-deoxyguanosine; CRP, C-reactive protein; BER, Base excision repair; HCC, Hepatocellular carcinoma; MDA, Malondialdehyde; 4-HNE, 4-hydroxynonenal; PUFAs, Polyunsaturated fatty acids; CL, Cardiolipin; ACC1, acetyl-CoA carboxylase 1; PUFA, Polyunsaturated fatty acid; NSCLC, Non-small cell lung cancer; ICIs, Immune checkpoint inhibitors; SOD, Superoxide dismutase; GPx, Glutathione peroxidase; CSC, Cancer stem cells; CRP/ALB, C-reactive protein/albumin; PGs, Prostaglandins; PGE2, Prostaglandin E2; FAP, Familial adenomatous polyposis; NSCLC, Non-small cell lung cancer; HDACs, Histone deacetylases; DCA, Deoxycholic acid; FXR, Farnesoid X receptor; LCA, Lithocholic acid; 4-HPR, N-(4-hydroxyphenyl)-retinamide; TrxR, Thioredoxin reductase; BCL-2, B-cell lymphoma-2; GSSG, Glutathione disulfide; TNBC, Triple-negative breast cancer; CAFs, Cancer-associated fibroblasts; NSAIDs, Non-steroidal anti-inflammatory drugs; DFS, Disease-free survival; PTGS2, Prostaglandin-endoper oxide synthase 2; PDAC, Pancreatic ductal adenocarcinoma; TNF, Tumor necrosis factor; M/P, Mitoxantrone/prednisone; CRPC, Castration-resistant prostate cancer; IFN-α, Interferon-α; M1, M1-type macrophages; M2, M2-type macrophages; N1, N1-type neutrophils; N2, N2-type neutrophils; MDSC, Myeloid-derived suppressor cells; DC, Dendritic cell; O2•^−^, Superoxide anion; OH•, Hydroxyl radical; NO2•, Nitrogen dioxide; RO•/ROO•, Alkoxyl/alkyl peroxyl, H_2_O_2_, Hydrogen peroxide; NO, Nitric oxide; HOCl, Hypochlorous acid.
